# Supplementary material for: Prevalence and impact of chronic dysglycaemia among patients with COVID-19 in Swedish intensive care units: a multicentre, retrospective cohort study
Source: BMJ Open. 2023 Sep 19;13(9):e071330. doi: 10.1136/bmjopen-2022-071330 (PMC10510869; doi:10.1136/bmjopen-2022-071330)
Supplement: Supplementary data [file bmjopen-2022-071330supp001.pdf]

Table S1: Post-hoc exploratory comparison between the subgroups for 90 days mortality and Renal replacement therapy

Subgroups	90 day mortality		Renal replacement therapy	
	N (%)	p	N (%)	p
No chronic dysglycemia vs Prediabetes	9/61 (14.7) vs 28/114 (24.5)	0.17	17/61 (27.8) vs 22/114 (19.2)	0.25
No chronic dysglycemia vs Unknown diabetes	9/61 (14.7) vs 12/60 (20.0)	0.48	17/61 (27.8) vs 11/60 (18.3)	0.28
No chronic dysglycemia vs Controlled diabetes	9/61 (14.7) vs 7/25 (28.0)	0.22	17/61 (27.8) vs 1/25 (4.0)	0.01
No chronic dysglycemia vs Uncontrolled diabetes	9/61 (14.7) vs 15/48 (31.2)	0.06	17/61(27.8) vs 8/48 (16.6)	0.25
Prediabetes vs Unknown diabetes	28/114 (24.5) vs 12/60 (20.0)	0.57	22/114 (19.2) vs 11/60 (18.3)	1
Prediabetes vs Controlled diabetes	28/114 (24.5) vs 7/25 (28.0)	0.79	22/114 (19.2) vs 1/ 25 (4.0)	0.07
Prediabetes vs Uncontrolled diabetes	28/114 (24.5) vs 15/48 (31.2)	0.43	22/114 (19.2) vs 8/48 (16.6)	0.8
Unknown diabetes vs Controlled diabetes	12/60 (20.0) vs 7/25 (28.0)	0.41	11/60 (18.3) vs 1/25 (4.0)	0.1
Unknown diabetes vs Uncontrolled diabetes	12/60 (20.0) vs 15/48 (31.2)	0.18	11/60 (18.3) vs 8/48 (16.6)	1
Controlled vs Uncontrolled diabetes	7/25 (28.0) vs 15/48 (31.2)	0.77	1/25 (4.0) vs 8/48 (16.6)	0.01
No chronic dysglycemia and prediabetes vs unknown and known diabetes	37/175 (21.1) vs 34/133 (25.5)	0.41	39/175 (22.2) vs 20/133 (15.0)	0.14

P values calculated with Fischer’s exact test

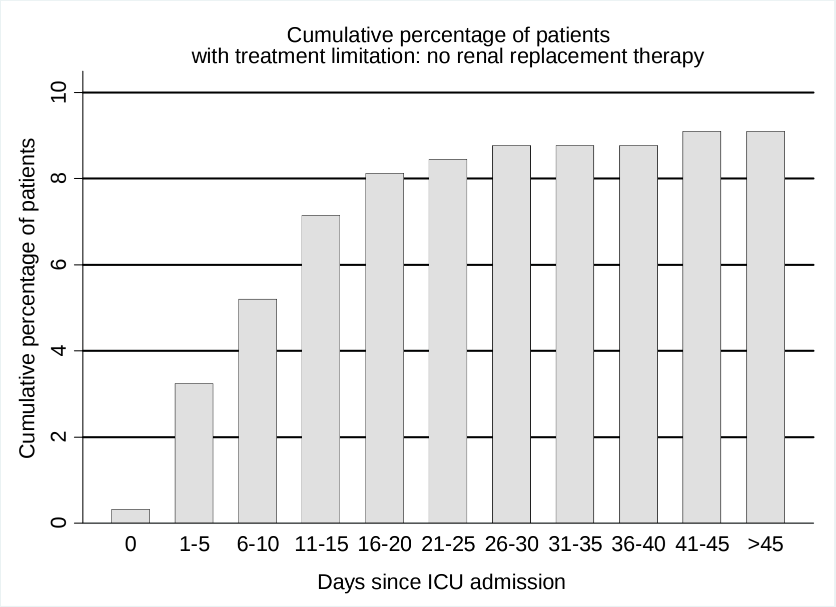

Figure S1. Cumulative percentage of patients with a treatment limitation: no renal replacement therapy. Day 0 (zero) denotes the day of admission to the intensive care unit.

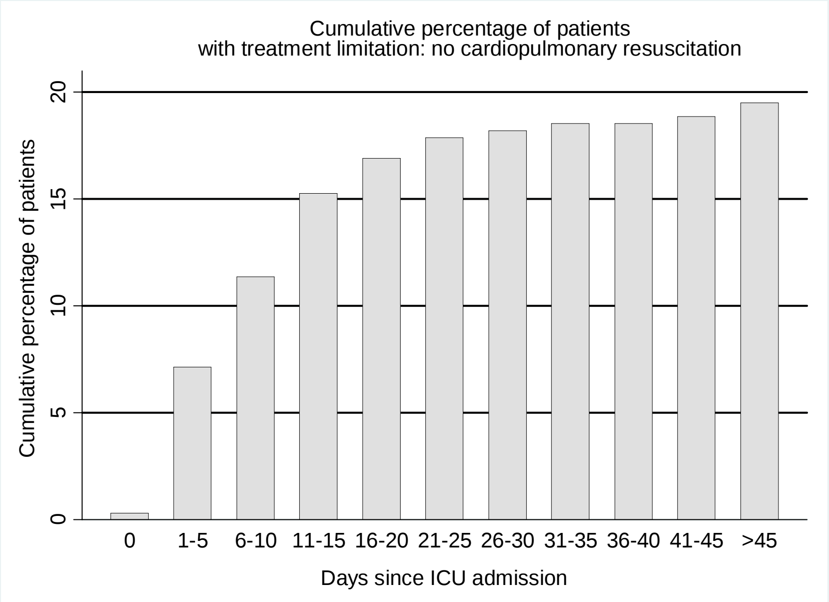

Figure S2. Cumulative percentage of patients with a treatment limitation: no cardiopulmonary resuscitation. Day 0 (zero) denotes the day of admission to the intensive care unit.

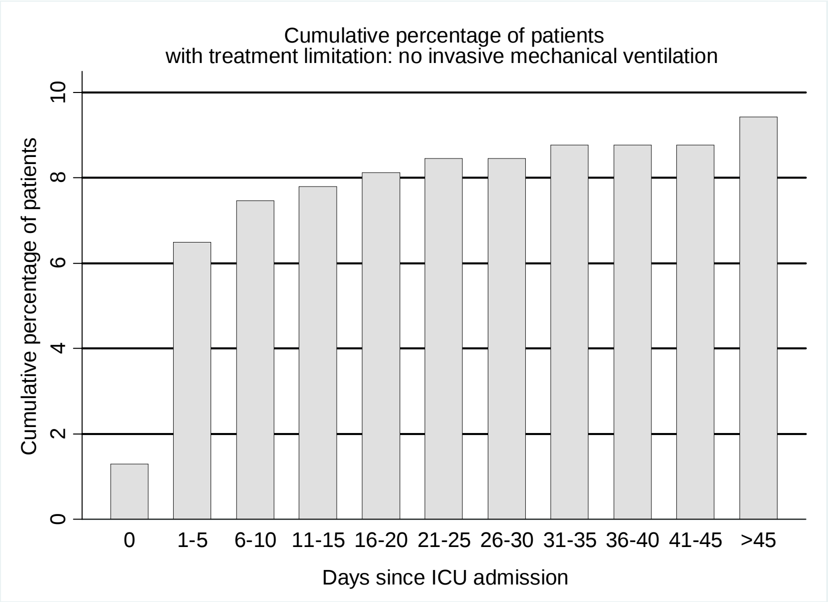

Figure S3. Cumulative percentage of patients with a treatment limitation: no invasive mechanical ventilation. Day 0 (zero) denotes the day of admission to the intensive care unit.

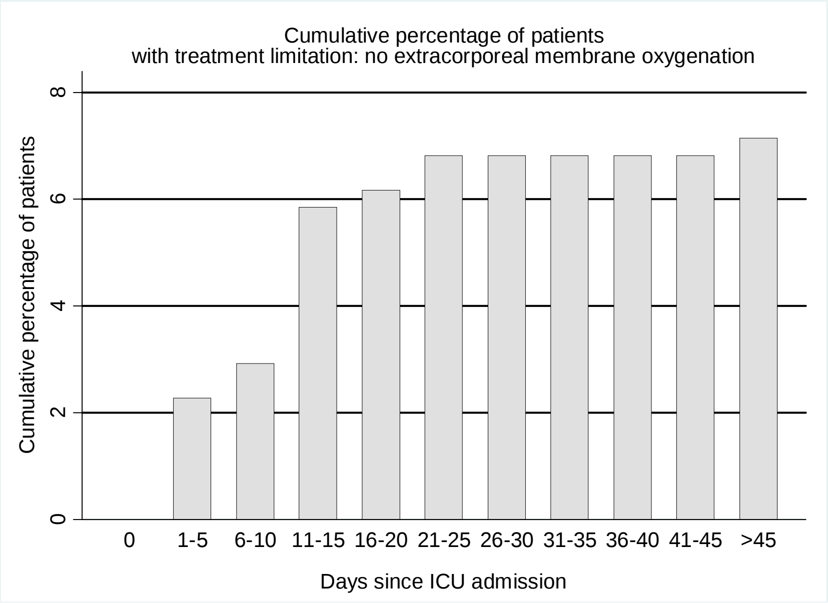

Figure S4. Cumulative percentage of patients with a treatment limitation: no extracorporeal membrane oxygenation. Day 0 (zero) denotes the day of admission to the intensive care unit.

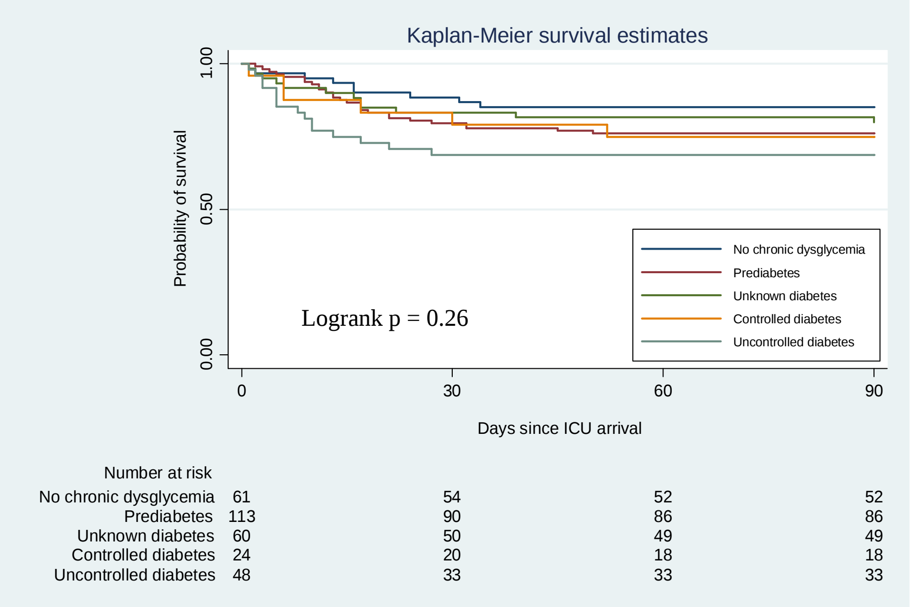

Figure S5. Probability of survival in the study groups
