# Supplementary material for: Prevalence and impact of chronic dysglycaemia among patients with COVID-19 in Swedish intensive care units: a multicentre, retrospective cohort study
Source: BMJ Open. 2023 Sep 19;13(9):e071330. doi: 10.1136/bmjopen-2022-071330 (PMC10510869; doi:10.1136/bmjopen-2022-071330)
Supplement: Author's manuscript [file bmjopen-2022-071330.draft_revisions.pdf]

BMJ Open is committed to open peer review. As part of this commitment we make the peer review history of every article we publish publicly available.

When an article is published we post the peer reviewers' comments and the authors' responses online. We also post the versions of the paper that were used during peer review. These are the versions that the peer review comments apply to.

The versions of the paper that follow are the versions that were submitted during the peer review process. They are not the versions of record or the final published versions. They should not be cited or distributed as the published version of this manuscript.

BMJ Open is an open access journal and the full, final, typeset and author-corrected version of record of the manuscript is available on our site with no access controls, subscription charges or pay-per-view fees (http://bmjopen.bmj.com).

If you have any questions on BMJ Open's open peer review process please email info.bmjopen@bmj.com

Chronic dysglycemia in critically ill patients with Covid-19 – a retrospective cohort study

Journal:	*BMJ Open*
Manuscript ID	bmjopen-2022-071330
Article Type:	Original research
Date Submitted by the Author:	23-Dec-2022

Complete List of Authors:	Balintescu, Anca; Karolinska Institute, Department of Clinical Science and Education, Södersjukhuset<br>Rysz, Susanne; Karolinska Institute, Department of Perioperative Medicine and Intensive Care<br>Hertz, Carl; Stockholm South General Hospital Anaesthesia Department ICU<br>Grip, Jonathan; Karolinska Institute, Department of Perioperative Medicine and Intensive Care<br>Cronhjort, Maria; Karolinska Institute, Department of Clinical Science and Education Södersjukhuset<br>Oldner, Anders; Karolinska Institute, Department of Perioperative Medicine and Intensive Care<br>Svensen, Christer; Karolinska Institute, Department of Clinical Science and Education Södersjukhuset<br>Mårtensson, Johan; Karolinska Institute, Department of Perioperative Medicine and Intensive Care

Keywords:	Adult intensive & critical care < ANAESTHETICS, COVID-19, DIABETES & ENDOCRINOLOGY, General diabetes < DIABETES & ENDOCRINOLOGY

SCHOLARONE™
Manuscripts

I, the Submitting Author has the right to grant and does grant on behalf of all authors of the Work (as defined in the below author licence), an exclusive licence and/or a non-exclusive licence for contributions from authors who are: i) UK Crown employees; ii) where BMJ has agreed a CC-BY licence shall apply, and/or iii) in accordance with the terms applicable for US Federal Government officers or employees acting as part of their official duties; on a worldwide, perpetual, irrevocable, royalty-free basis to BMJ Publishing Group Ltd ("BMJ") its licensees and where the relevant Journal is co-owned by BMJ to the co-owners of the Journal, to publish the Work in this journal and any other BMJ products and to exploit all rights, as set out in our licence.

The Submitting Author accepts and understands that any supply made under these terms is made by BMJ to the Submitting Author unless you are acting as an employee on behalf of your employer or a postgraduate student of an affiliated institution which is paying any applicable article publishing charge ("APC") for Open Access articles. Where the Submitting Author wishes to make the Work available on an Open Access basis (and intends to pay the relevant APC), the terms of reuse of such Open Access shall be governed by a Creative Commons licence – details of these licences and which Creative Commons licence will apply to this Work are set out in our licence referred to above.

Other than as permitted in any relevant BMJ Author's Self Archiving Policies, I confirm this Work has not been accepted for publication elsewhere, is not being considered for publication elsewhere and does not duplicate material already published. I confirm all authors consent to publication of this Work and authorise the granting of this licence.

For peer review only

Chronic dysglycemia in critically ill patients with Covid-19 – a retrospective cohort study

Anca Balintescu, MD[1, 2]; Susanne Rysz, MD[3, 4]; Carl Hertz, MD[2]; Jonathan Grip, MD, PhD[3, 5]; Maria Cronhjort, MD, PhD[1, 2]; Anders Oldner MD, PhD[3, 6]; Christer Svensen MD, PhD[1, 2]; Johan Mårtensson, MD, PhD[3, 6]

[1] Department of Clinical Science and Education Karolinska Institute, Unit of Anesthesiology and Intensive Care, South General Hospital, Sjukhusbacken 10, 11883 Stockholm, Sweden

[2] Department of Anesthesia and Intensive Care, South General Hospital, Sjukhusbacken 10, 11883 Stockholm, Sweden

[3] Department of Perioperative Medicine and Intensive Care, Karolinska University Hospital, Norrbacka S2:05, SE-17176 Stockholm, Sweden

[4] Department of Medicine, Solna, Karolinska Institute, Stockholm, Sweden

[5] Department of Clinical Science, Intervention and Technology, Karolinska Institute, Stockholm, Sweden

[6] Department of Physiology and Pharmacology, Section of Anaesthesia and Intensive Care, Karolinska Institutet, Norrbacka S2:05, SE-17176 Stockholm, Sweden

Corresponding Author:

Anca Balintescu, ANOPIVA, Södersjukhuset, Sjukhusbacken 10, 11883 Stockholm, Sweden. Phone: +46 (0)722 7023 83. Fax: +46 (0)8-616 22 08. Email: anca.balintescu@ki.se

Keywords: HbA1c, ICU, Covid-19, prevalence, glycemic control, diabetes

For peer review only - http://bmjopen.bmj.com/site/about/guidelines.xhtml

Abstract

Objective: Using glycated hemoglobin A1c (HbA1c) screening, we aimed to determine the prevalence of chronic dysglycemia (prediabetes or diabetes) among patients with Covid-19 admitted to the intensive care unit (ICU). Additionally, we aimed to explore the association between chronic dysglycemia and clinical outcomes related to ICU stay.

Design: Multicenter prospective observational study

Setting: ICUs in three hospitals in Stockholm, Sweden

Participants: Covid-19 patients admitted to the ICU between 5 March and 13 August 2020 with available HbA1c at admission. Chronic dysglycemia was determined based on previous history of diabetes and HbA1c.

Primary and secondary outcomes measures: Primary outcome was the actual prevalence of chronic dysglycemia (prediabetes, unknown diabetes, or known diabetes) among Covid-19 patients. Secondary outcome was the association of chronic dysglycemia with 90-day mortality, ICU length of stay, duration of invasive mechanical ventilation (IMV), and renal replacement therapy (RRT) use, accounting for treatment selection bias.

Results: A total of 308 patients with available admission HbA1c were included. Chronic dysglycemia prevalence assessment was restricted to 206 patients admitted to three ICUs in which HbA1c was measured on all admitted patients. Chronic dysglycemia was present in 82.0% (95% CI 76.1%-87.0%) of patients, with prediabetes present in 40.2% (95% CI 33.5%-47.3%), unknown diabetes in 20.9% (95% CI 15.5%-27-1%), well-controlled diabetes in 7.8% (95% CI 4.5%-12.3%), and uncontrolled diabetes in 13.1% (95% CI 8.8%-18.5%). All patients with available HbA1c were included for the analysis of the relationship between chronic

For peer review only - http://bmjopen.bmj.com/site/about/guidelines.xhtml

dysglycemia and secondary outcomes. We found no independent association between chronic dysglycemia and 90-day mortality, ICU length of stay, duration of IMV or RRT use. Risk estimates remained virtually unchanged after excluding patients with specific treatment limitations.

Conclusions: In our cohort of critically ill Covid-19 patients, the prevalence of chronic dysglycemia was 82%. We found no association between chronic dysglycemia and clinical outcomes.

Strengths and limitations of this study

- Presents prevalence of chronic dysglycemia in an ICU population with Covid-19 based on additional quantification of admission HbA1c
- Actual prevalence of chronic dysglycemia calculation in all ICU admitted patients, reducing the risk of ascertainment bias
- Treatment limitations were considered in the analysis of clinical outcomes, thereby reducing the risk of treatment selection bias.
- We lack data on glycemic control during ICU stay, that might have influenced clinical outcomes

Background

Diabetes has been identified as a frequent comorbidity in patients with Covid-19, with a prevalence ranging from 7.4% to 34.3% among those requiring hospitalization [1-3]. A meta-analysis published in April 2020 found diabetes to be the second most

For peer review only - http://bmjopen.bmj.com/site/about/guidelines.xhtml

frequent comorbidity in patients with Covid-19 admitted to the intensive care unit (ICU) [4]. Furthermore, Covid-19 patients with diabetes appear to have a significantly higher risk of ICU admission and worse prognosis than Covid-19 patients without diabetes [4-6]. Particularly, a glycated hemoglobin A1c (HbA1c) level above 7% (53 mmol/mol) was identified as risk factor for ICU admission [7].

Recent data also indicates that diabetes is associated with worse prognosis among ICU patients with Covid-19 [8]. However, these studies did not include HbA1c measurements to identify patients with prediabetes or previously undiagnosed diabetes. This is an important limitation since both prediabetes and diabetes is considerably under-diagnosed both in the community [9] and in the ICU [10]. Additionally, a history of diabetes diagnosis and HbA1c at ICU admission, measured in consecutively admitted patients, is important in determining the true prevalence of chronic dysglycemia in the critically ill Covid-19 population. Finally, information about limitations of life-sustaining treatment were not considered in previous outcome analyses. This is unfortunate since the presence of such limitations may introduce treatment selection bias.

We therefore conducted a multicenter observational study using quantification of HbA1c and information about diabetes history to determine the actual prevalence of chronic dysglycemia (prediabetes, unknown diabetes, or known diabetes) among Covid-19 patients admitted to ICU. In addition, we aimed to explore the relationship of chronic dysglycemia with 90-day mortality, ICU length of stay, duration of invasive mechanical ventilation (IMV) and severe acute kidney injury requiring renal replacement therapy (RRT) accounting for treatment selection bias. We hypothesised that prevalence of chronic dysglycemia in Covid-19 patients admitted to the ICU exceeds the prevalence of chronic dysglycemia in the non Covid-19 critically ill

For peer review only - http://bmjopen.bmj.com/site/about/guidelines.xhtml

population. Moreover, we hypothesised that such chronic dysglycemia would be associated with worse clinical outcomes during ICU stay in patients with Covid-19.

Material and Methods

The study was approved by the Swedish Ethical Review Authority (approval number 2020-01302, amendment 2020-02890) with a waiver of informed consent. The study was performed in accordance with the Helsinki Declaration and reported in conformity with the STROBE statement [11]

Patient and Public Involvement statement: The study is based on data that was collected during the ongoing Covid-19 pandemic in a quality register. No intervention was applied to the individual patient. The public and patients were not involved in the design of the study. Results are to be disseminated to the public and scientific community through publication in peer-reviewed journal with open access.

Study design

We conducted a multicenter, prospective observational study of adult (≥18 years) patients with a positive polymerase chain reaction (PCR) for severe acute respiratory syndrome coronavirus 2 (SARS-CoV-2) admitted to ten ICUs in three hospitals in Stockholm, Sweden between March 5th and August 13th, 2020 (first wave). We excluded patients without HbA1c obtained on admission to the ICU, patients in the third trimester of pregnancy and patients with a primary admission diagnosis other than Covid-19. In patients with multiple ICU admissions, only the first admission was considered. All included patients were assessed in the outcome analyses.

Assessment of chronic dysglycemia prevalence was restricted to a nested cohort of

For peer review only - http://bmjopen.bmj.com/site/about/guidelines.xhtml

patients from ICUs in which HbA1c measurement was included in the routine laboratory panel performed on all consecutive admissions. In the prevalence analysis, we therefore excluded patients with available HbA1c who were admitted to ICUs in which HbA1c was measured only at the discretion of the treating clinicians.

Data collection

HbA1c was measured in whole blood at ICU admission using the VARIANT II TURBO Hemoglobin Testing System analyzer (Bio-Rad Laboratories GmBH) and was reported in mmol/mol (IFCC calibrated) and in %. HbA1c was measured as part of routine care in three ICUs and at the discretion of the treating clinician in seven ICUs. We collected information on demographics, comorbidity, chronic medication, HbA1c value, mortality and decision regarding limitation of life-sustaining care from the patients' medical records (Take Care [CompuGroup Medical, Koblenz, Germany]). International Classification of Disease (ICD) 10 codes were used to identify comorbidity and previous history of diabetes. Additionally, data regarding known diabetes diagnosis was extracted manually from the patients' medical records. Data on body mass index (BMI), Simplified Acute Physiology Score (SAPS) 3, ICU length of stay, duration of IMV and RRT were collected from the ICU electronic patient data management system Clinisoft (GE, Barringgton, IL).

Prediabetes and Diabetes definitions

Prediabetes and diabetes were diagnosed based on two complementary methods; level of HbA1c at admission and previous medical history of diabetes, and categorized into five groups:

(1) no diabetes (HbA1c <42 mmol/mol [6.0%] and no history of diabetes)

For peer review only - http://bmjopen.bmj.com/site/about/guidelines.xhtml

(2) prediabetes (HbA1c 42-47 mmol/mol [6.0-6.4%] and no history of diabetes)

(3) unknown diabetes (HbA1c ≥48 mmol/mol [6.9 %] and no history of diabetes)

(4) controlled diabetes (HbA1c <52 mmol/mol [6.9 %] and previous history of diabetes)

(5) uncontrolled diabetes (HbA1c ≥52 mmol/mol [6.9 %] and previous history of diabetes).

Cut off values for HbA1c according to the World Health Organization were used [12]. Individuals in group (2), (3), (4) and (5) were considered to have chronic dysglycemia compared to those in group (1) labeled "no chronic dysglycemia".

Outcomes

The primary outcome was the prevalence of chronic dysglycemia. Secondary outcomes included 90-day mortality, ICU length of stay, duration of IMV, and RRT use.

Statistical Analysis

We analyzed data using STATA version 12.1 (Stata Corp., College Station, Texas). Categorical data is presented as numbers and percentages and compared using the chi-square test or Fisher's exact test. Continuous data is summarized as median with interquartile range (IQR) and compared using the Mann-Whitney U test (for two groups) or the Kruskal Wallis test (for multiple groups). The prevalence of chronic dysglycemia (primary outcome) was presented as percentages with 95% confidence intervals (CI). We displayed time to death within 90 days using Kaplan-Meier curves. Survival curves were compared using a log-rank test. We used multivariable Cox regression analysis to assess the association between chronic dysglycemia and 90-

For peer review only - http://bmjopen.bmj.com/site/about/guidelines.xhtml

day mortality. We used multivariable linear regression analysis to assess the association with ICU length of stay and duration of IMV. Both these outcomes were found to be well approximated by log-normal distributions and were therefore log-transformed before analysis with results presented as geometric means (95% CI). We used multivariable logistic regression analysis to assess the association with RRT use, before and after excluding patients with RRT as a treatment limitation. All regression models were adjusted for the following predetermined confounders: SAPS 3, age and sex. A two-sided P-value <0.05 was considered statistically significant.

Results

Patients

A total of 584 patients with positive SARS-CoV-2 test were admitted to the study ICUs during the study period. We excluded 225 patients without available HbA1c, six pregnant patients, 16 readmissions and 29 patients without symptoms associated with Covid-19. Therefore, we included 308 patients with available HbA1c for outcome analysis. Among those 308 patients, 206 consequently admitted patients in which HbA1c was included in the admission routine laboratory panel were used for prevalence calculation (Figure 1). Baseline characteristics and treatment limitations of the entire study population are detailed in Table 1.

Table 1. Baseline characteristics and treatment limitations

		Chronic dysglycemia				
**Characteristic**	**No chronic dysglycemia**	**Prediabetes**	**Unknown diabetes**	**Controlled diabetes**	**Uncontrolled diabetes**	**P[e]**
No. (%)	61 (19.8)	114 (37.0)	60 (19.4)	25 (8.11)	48 (15.5)	
Age, years	57 (51,63)	61 (53, 68)	60 (52, 68)	63 (57, 71)	62 (55,69)	0.02
Male sex	48 (78.6)	92 (80.7)	47 (78.3)	21 (84.0)	36 (75.0)	0.90
Body mass index[a], kg/m^2	27 (25, 32)	27 (25, 30)	28 (25, 31)	29 (26, 32)	30 (26, 33)	0.97
HbA1c, mmol/mol	39 (36, 40)	44 (43, 46)	51 (49, 57)	47 (44, 49)	70 (61, 81)	<0.001
Diabetes treatment						
Diet only				6 (24.0)	2 (4.1)	
OAD only				17 (68)	19 (39.5)	

For peer review only - http://bmjopen.bmj.com/site/about/guidelines.xhtml

Insulin only				1 (4.0)	12 (25.0)	
OAD+Insulin				1 (4.0)	15 (31.2)	
Comorbidity						
Hypertension	18 (29.5)	40 (35.0)	23 (38.3)	16 (64.0)	34 (70.8)	0.02
Heart failure	6 (9.8)	5 (4.3)	6 (10.0)	0 (0.0)	3 (6.2)	0.24
Previous myocardial infarction	2 (3.2)	4 (3.5)	6 (10.0)	0 (0.0)	7 (14.5)	0.38
Chronic kidney disease	4 (6.5)	13 (11.4)	7 (11.6)	6 (24.0)	11 (22.9)	0.09
Liver disease	2 (3.2)	4 (3.5)	1 (1.6)	1 (4.0)	1 (2.0)	1.00
Any malignancy	0 (0.0)	8 (7.0)	2 (3.3)	2 (8.0)	4 (8.3)	0.04
Astma/COPD	13 (21.3)	20 (17.5)	14 (23.3)	5 (20.0)	9 (18.7)	0.72
SAPS 3[b]	53 (48, 60)	55 (49, 60)	57 (52, 62)	59 (52, 63)	56 (52, 69)	0.18
Chronic drug use						
Corticosteroids[c]	5 (8.20)	16 (14.04)	8 (13.3)	4 (16.0)	6 (12.5)	0.24
Immunosuppressive therapy[d]	1 (1.6)	8 (7.0)	3 (5.0)	1 (4.0)	1(2.0)	0.31
Treatment limitations[f]						
Any limitation	14 (22.9)	19 (16.6)	13 (21.6)	8 (32.0)	13 (27.0)	0.80
No RRT	5 (8.2)	7 (6.1)	5 (8.3)	5 (20.0)	6 (12.5)	0.78
No IMV	6 (9.8)	10 (8.7)	4 (6.6)	4 (16.0)	6 (12.5)	0.97
No CPR	9 (14.7)	19 (16.6)	12 (20.0)	8 (32.0)	12 (25.0)	0.29
No ECMO	7 (11.4)	3 (2.6)	5 (8.3)	3 (12.0)	3 (6.2)	0.10
Palliative care[g]	1 (1.6)	18 (16.5)	7 (12.2)	4 (16.6)	5 (10.8)	0.03

Data are n (%) or median (interquartile range)
HbA1c, glycated hemoglobin A1c; OAD, oral hypoglycemic agent; COPD, Chronic obstructive pulmonary disease; SAPS, Simplified Acute Physiology Score, 2 missing values (306 patients with data); RRT, renal replacement therapy; IMV, invasive mechanical ventilation; CPR, cardiopulmonary resuscitation
[a]Missing data in 15 patients (293 patients with data)
[b]Missing data in 2 patients (306 patients with data)
[c]Systemic or inhalatory corticosteroids
[d]Immunosuppressive therapy was defined as: treatment with Metotrexate, Azatioprin, Ciklosporin, Tracolimus, Infliximab
[e]P values for the comparison between no chronic dysglycemia and chronic dysglycemia
[f] Decision taken any time during ICU stay
[g]Decision to go over to palliative care taken during ICU stay

Patients with chronic dysglycemia were older, were more likely to have hypertension, malignancy and/or chronic kidney disease, and had higher SAPS 3 than patients without chronic dysglycemia. Overall, 14 (22.9%) patients in the no chronic dysglycemia group and 53 (21.5%) patients in the chronic dysglycemia group received one or more limitations of life-supporting therapies during their ICU stay. “No Cardiopulmonary resuscitation (CPR)” was the most common treatment limitation. We observed the highest proportion of limitations among patients with known (controlled or uncontrolled) diabetes. Decision to switch to palliative care was made in 1 (1.6%) patient in the no chronic dysglycemia group and 34 (13.8%) patients in the chronic dysglycemia group (P=0.03). Cumulative percentage of treatment limitations relative ICU admission is displayed in Figures S1-S4.

For peer review only

For peer review only - http://bmjopen.bmj.com/site/about/guidelines.xhtml

Primary outcome

In the nested cohort of 206 consecutive patients with available HbA1c, 169 (82.0%; 95% CI 76.1%-87.0%) were diagnosed with chronic dysglycemia. Prediabetes was present in 83 (40.2%, 95% CI 33.5%-47.3%), unknown diabetes in 43 (20.9%, 95% CI 15.5%-27-1%), well-controlled diabetes in 16 (7.8%, 95% CI 4.5%-12.3%), and uncontrolled diabetes in 27 (13.1%, 95% CI 8.8%-18.5%) patients (Figure 2).

Secondary outcomes

Nine (14.7%) patients in the no chronic dysglycemia group and 62 (25.1%) patients in the chronic dysglycemia group died within 90 days (P=0.08) (Table 2, Figure 3 and Figure S5). ICU length of stay and duration of IMV were similar in the two groups. IMV was delivered to 42 (68.8%) patients without chronic dysglycemia and to 187 (75.7%) patients with chronic dysglycemia (P=0.27). RRT was delivered to 17 (27.9%) no chronic dysglycemia patients and 42 (17.0%) chronic dysglycemia patients (P=0.06) (Table 2 and Table 3).

Table 2. Secondary outcomes

		Chronic dysglycemia				
**Outcomes**	**No chronic dysglycemia (n = 61)**	**Prediabetes (n = 114)**	**Unknown diabetes (n =60)**	**Controlled diabetes (n = 25)**	**Uncontrolled diabetes (n = 48)**	**P[a]**
90-day mortality, n (%)	9 (14.7)	28 (24.5)	12 (20.0)	7 (28.0)	15 (31.2)	0.08
ICU length of stay, days	9 (4, 25)	14 (6, 24)	13 (6, 28)	8 (5, 21)	11 (7, 22)	0.69
Invasive mechanical ventilation, days	16 (8, 29)	14 (10, 23)	15 (10, 27)	15 (6, 21)	14 (9, 22)	0.60
Renal replacement therapy, n (%)	17 (27.9)	22 (19.3)	11 (18.3)	1 (4.0)	8 (16.6)	0.06

Data are n (%) or median (interquartile range)
ICU lengths of stay (4 missing values because of transfer to other hospital)
[a] P values for the comparison between no chronic dysglycemia and chronic dysglycemia

For peer review only - http://bmjopen.bmj.com/site/about/guidelines.xhtml

Table 3. Multivariable regression analyses showing the association of chronic dysglycemia (versus no chronic dysglycemia) with secondary outcomes

Outcome measure	No Chronic Dysglycemia	Chronic Dysglycemia	Adjusted Risk Estimate (95% CI)[a]	Statistical test	P Value
90-day mortality, n (%)	9/61 (14.7)	62/247 (25.1)	1.61 (0.79 to 3.26)	Cox regression	0.18
ICU length of stay, days					
All patients	9 (4, 25)	13 (6, 23)	1.06 (0.78 to 1.43)	Linear regression	0.70
ICU survivors[b]	9 (5, 27)	14 (7, 24)	1.04 (0.76 to 1.43)	Linear regression	0.75
Invasive mechanical ventilation duration, days					
All patients[c]	16 (8, 29)	14 (10, 23)	0.92 (0.68 to 1.23)	Linear regression	0.58
ICU survivors[d]	16 (8, 30)	15 (10, 23)	0.93 (0.70 to 1.23)	Linear regression	0.61
Renal replacement therapy, n (%)					
All patients	17/61 (27.9)	42/247 (17.0)	0.52 (0.26 to 1.02)	Logistic regression	0.06
Patients without treatment limitation as no RRT	17/57 (29.8)	42/224 (18.8)	0.52 (0.26 to 1.04)	Logistic regression	0.10

[a]Multivariable models were adjusted for Simplified Acute Physiology Score (SAPS) 3, age and sex.
[b]ICU length of stay in ICU survivors, 260 observations
[c]Invasive mechanical ventilation duration, 227 observations
[d]Invasive mechanical ventilation duration in ICU survivors, 189 observations

On multivariable regression analysis we observed a trend towards higher mortality (adjusted HR 1.61, 95% CI 0.79-3.26, P=0.18) and lower RRT use (adjusted OR 0.52, 95% CI 0.26-1.02, P=0.06) in patients with chronic dysglycemia (Table 3). The association with RRT use remained virtually unchanged after exclusion of patients with "No RRT" as treatment limitation.

Discussions

Key findings

We performed a multicenter observational investigation to determine the prevalence of chronic dysglycemia and its impact on clinical outcomes among Covid-19 patients admitted to ICU. Using available information about the patients' diabetic status in combination with routine HbA1c assessment, we found that 82% had chronic

For peer review only - http://bmjopen.bmj.com/site/about/guidelines.xhtml

dysglycemia with two thirds having either prediabetes or undiagnosed diabetes. We observed a trend towards increased 90-day mortality in patients with chronic dysglycemia, with the highest mortality (31%) observed among those with uncontrolled diabetes. Conversely, the proportion of patients receiving RRT was lower among patients with chronic dysglycemia even when patients without "No RRT" as treatment limitation were considered separately. We found no association of chronic dysglycemia with ICU length of stay or duration of IMV.

Relationship with previous studies

A global meta-analysis of more than 16000 ICU patients with Covid-19 suggests a pooled prevalence of known diabetes between 23-31% [13], close to the observed prevalence in our study (21%). However, few studies have used additional HbA1c measurements to assess the actual prevalence of chronic dysglycemia, including prediabetes and undiagnosed diabetes. One such ICU study from Austria found a prevalence of chronic dysglycemia of 85%, which is in close agreement with our findings [14]. However, the Austrian study did not assess consecutive patients and may therefore be prone to selection bias.

Our findings indicate that chronic dysglycemia is more common in Covid-19 patients than in ICU patients with other admission diagnoses. In fact, in a pre-Covid-19 cohort of general ICU patients we found a corresponding dysglycemia prevalence of 33% [10]. The relationship between severe SARS-CoV-2 infection and dysglycemia has different potential explanations. SARS-CoV-2 enters cells in various organs, including the pancreas, via angiotensin-converting enzyme II (ACE2). As ACE2 is involved in regulating pancreatic beta-cell function, a link between SARS-CoV-2 infection and beta-cell dysfunction and diabetes development has been suggested [15].

For peer review only - http://bmjopen.bmj.com/site/about/guidelines.xhtml

Interestingly, the prevalence of elevated HbA1c below the diabetes diagnostic threshold (prediabetes) was markedly higher in our Covid-19 cohort than in our previous pre-Covid-19 cohort (40% vs 9%). It is possible that the duration of Covid-19 symptoms before ICU admission (typically ten days in the literature [16]) was sufficient to trigger new onset hyperglycemia with mildly elevated HbA1c.

In addition to the above speculations about SARS-CoV-2 as a *cause* of dysglycemia, there is also evidence suggesting that patients with preexisting dysglycemia are prone to a more severe course of Covid-19. For example, some studies have shown that hospitalized SARS-CoV-2 positive with prediabetes, unknown diabetes, and known poorly controlled diabetes are at increased risk of SARS-CoV-2 associated respiratory failure requiring intensive care [17]. A higher burden of comorbidities, hyperglycemia *per se*, and chronic low-grade inflammation in diabetes may explain this observation[18].

Whether chronic dysglycemia is associated with worse outcomes among Covid-19 patients admitted to ICU remains uncertain. A multicenter study from France including 410 ICU patients with Covid-19, found no association between the severity of dysglycemia and tracheal intubation and/or death within 7 days of admission in patients with diabetes than in those without diabetes [19]. This is in accordance with the findings of our study. In contrast, others found higher mortality in the subgroup of mechanically ventilated patients with diabetes [14].

We previously demonstrated an independent association between chronic dysglycemia and need for RRT in critically ill non-Covid-19 patients [10]. This association was, however, not found in the present study. In fact, we observed a higher proportion of patients requiring RRT among our patients without chronic dysglycemia. Exclusion of patients “not for RRT”, did not substantially alter this

For peer review only - http://bmjopen.bmj.com/site/about/guidelines.xhtml

finding. Importantly, limitations in life-sustaining care were more common in the known diabetes groups (well controlled and uncontrolled diabetes) than in all other groups. We cannot exclude the possibility that patients with severe acute or chronic kidney injury did not reach the ICU because of treatment limitation decisions made at hospital arrival or on the medical ward. This might have influenced the number of patients with kidney injury reaching the ICU, affecting predominantly patients with chronic dysglycemia, as they are usually older and have multiple comorbidities.

Strengths and limitations

Our study has several strengths. It is the first to assess the prevalence of chronic dysglycemia in an ICU population with Covid-19 based on additional quantification of admission HbA1c. This approach reduced bias due to events that would have influenced HbA1c values obtained before ICU admission. Additionally, we restricted the prevalence assessment to a cohort of patients who were admitted to ICUs where HbA1c was part of the routine laboratory panel, thereby reducing the risk of ascertainment bias. Furthermore, we considered treatment limitations in our analysis of clinical outcomes, thereby reducing the risk of treatment selection bias. Finally, we included patients admitted to ten ICUs in three University hospitals, thus providing a degree of external validity for applying our findings to similar settings.

Our study has limitations. We lack data on conditions and treatment that might have influenced admission HbA1c, such as haemoglobinopathies and blood transfusion before ICU admission. In addition, we lack information about glycemic control during intensive care, which might have modified clinical outcomes.

Conclusion

For peer review only - http://bmjopen.bmj.com/site/about/guidelines.xhtml

In our multicenter cohort of Covid-19 patients admitted to the ICU, HbA1c screening diagnosed chronic dysglycemia in four out of five patients with the majority having either prediabetes or previously undiagnosed diabetes. Chronic dysglycemia was not significantly associated with mortality, ICU length of stay, duration of invasive mechanical ventilation or renal replacement therapy use. These findings indicate that chronic dysglycemia may be a risk factor for severe Covid-19. However, Covid-19 prognosis in the ICU does not appear to be modified by chronic dysglycemia.

For peer review only

Acknoledgments

Author Contributions: Anca Balintescu, Johan Mårtensson, Maria Cronhjort, Christer Svensen, Anders Oldner, Jonathan Grip: Conceptualization, Methodology, Software, reviewing and Editing

Anca Balintescu, Susanne Rysz, Carl Hertz: Data curation

Anca Balintescu, Johan Mårtensson: Writing- Original draft preparation.

Funding A.B. and J.M. were supported by Region Stockholm (clinical research appointment and ALF project grants), grant number 580282.

A non-peer-reviewed version of this article has been a part of a publicly defended PhD thesis ISBN 978-91-8016-719-2 and was submitted to Karolinska Institute Open Archive, available at https://openarchive.ki.se/xmlui/handle/10616/48203 on 19 September 2022.

Competing interest: The authors have no competing interest relevant to this work.

Data availability: The data that support the findings of this study are available from the corresponding author upon reasonable request.

Ethics Approval Statement: The study was approved by the Swedish Ethical Review Authority (approval number 2020-01302).

References

[1] Yang J, Zheng Y, Gou X, Pu K, Chen Z, Guo Q, et al. Prevalence of comorbidities and its effects in patients infected with SARS-CoV-2: a systematic review and meta-analysis. International Journal of Infectious Diseases 2020;94:91-5. doi:10.1016/j.ijid.2020.03.017

[2] Liu Y, Lu R, Wang J, Cheng Q, Zhang R, Zhang S, et al. Diabetes, even newly defined by HbA1c testing, is associated with an increased risk of in-hospital death in adults with COVID-19. BMC Endocrine Disorders 2021;21(1):1-10. doi:10.1186/s12902-021-00717-6

[3] Guan W-j, Liang W-h, Zhao Y, Liang H-r, Chen Z-s, Li Y-m, et al. Comorbidity and its impact on 1590 patients with COVID-19 in China: a nationwide analysis. European Respiratory Journal 2020;55(5):2000547. doi:10.1183/13993003.00547-2020

[4] Roncon L, Zuin M, Rigatelli G, Zuliani G. Diabetic patients with COVID-19 infection are at higher risk of ICU admission and poor short-term outcome. J Clin Virol 2020;127:104354. doi:10.1016/j.jcv.2020.104354

[5] Zhou F, Yu T, Du R, Fan G, Liu Y, Liu Z, et al. Clinical course and risk factors for mortality of adult inpatients with COVID-19 in Wuhan, China: a retrospective cohort study. The Lancet 2020;395. doi:10.1016/S0140-6736(20)30566-3

[6] Guo W, Li M, Dong Y, Zhou H, Zhang Z, Tian C, et al. Diabetes is a risk factor for the progression and prognosis of COVID-19. Diabetes/metabolism research and reviews 2020:e3319 doi: 10.1002/dmrr.3319.

[7] Lei M, Lin K, Pi Y, Huang X, Fan L, Huang J, et al. Clinical Features and Risk Factors of ICU Admission for COVID-19 Patients with Diabetes. Journal of Diabetes Research 2020:5237840. doi:10.1155/2020/5237840

[8] Grasselli G, Greco M, Zanella A, Albano G, Antonelli M, Bellani G, et al. Risk Factors Associated With Mortality Among Patients With COVID-19 in Intensive Care Units in Lombardy, Italy. JAMA internal medicine 2020;180(10):1345-55. doi:10.1001/jamainternmed.2020.3539

[9] Beagley J, Guariguata L, Weil C, Motala AA. Global estimates of undiagnosed diabetes in adults. Diabetes Research and Clinical Practice 2014;103(2):150-60. doi:10.1016/j.diabres.2013.11.001

[10] Balintescu A, Palmgren I, Lipcsey M, Oldner A, Larsson A, Cronhjort M, et al. Prevalence and impact of chronic dysglycemia in intensive care unit patients-A retrospective cohort study. Acta Anaesthesiol Scand 2021;65(1):82-91. doi:10.1111/aas.13695

[11] Erik von Elm DGA, Matthias Egger, Stuart J. Pocock, Peter C. Gøtzsche, and Jan P. Vandenbroucke. The Strengthening the Reporting of Observational Studies in Epidemiology (STROBE) Statement: Guidelines for Reporting Observational Studies. Annals of Internal Medicine 2007;147(8):573-7. doi:10.7326/0003-4819-147-8-200710160-00010 %m 17938396

[12] Organisation WH. Use of Glycated Haemoglobin (HbA1c) in the Diagnosis of Diabetes Mellitus, https://www.who.int/publications/i/item/use-of-glycated-haemoglobin-(-hba1c)-in-diagnosis-of-diabetes-mellitus; [accessed 07 Fenruary 2022.

[13] Tan E, Song J, Deane AM, Plummer MP. Global Impact of Coronavirus Disease 2019 Infection Requiring Admission to the ICU: A Systematic Review and Meta-analysis. Chest 2021;159(2):524-36. doi:10.1016/j.chest.2020.10.014

[14] Klein SJ, Mayerhöfer T, Fries D, Preuß Hernández C, Joannidis M, Collaborators, et al. Elevated HbA1c remains a predominant finding in severe COVID-19 and may be associated with increased mortality in patients requiring mechanical ventilation. Critical Care 2021;25(1):1-4. doi:10.1186/s13054-021-03730-2

For peer review only - http://bmjopen.bmj.com/site/about/guidelines.xhtml

[15] Memon B, Abdelalim EM. ACE2 function in the pancreatic islet: Implications for relationship between SARS-CoV-2 and diabetes. Acta Physiologica 2021;233(4):1-13. doi:10.1111/apha.13733

[16] Larsson E, Brattstrom O, Agvald-Ohman C, Grip J, Jalde FC, Stralin K, et al. Characteristics and outcomes of patients with COVID-19 admitted to ICU in a tertiary hospital in Stockholm, Sweden. Acta anaesthesiologica Scandinavica 2021;65(1):76-81. doi:10.1111/aas.13694

[17] Rysz S, Jonsson Fagerlund M, Rimes-Stigare C, Larsson E, Campoccia Jalde F, Mårtensson J. Chronic dysglycemia and risk of SARS-CoV-2 associated respiratory failure in hospitalized patients. Acta anaesthesiologica Scandinavica 2022;66(1):48-55. doi:10.1136/bjsports-2021-104080.;

[18] Landstra CP, de Koning EJP. COVID-19 and Diabetes: Understanding the Interrelationship and Risks for a Severe Course. Frontiers in endocrinology 2021;12:649525. doi:10.3389/fendo.2021.649525

[19] Cariou B, Hadjadj S, Wargny M, Pichelin M, Al-Salameh A, Allix I, et al. Phenotypic characteristics and prognosis of inpatients with COVID-19 and diabetes: the CORONADO study. Diabetologia 2020;63(8):1500-15. doi:10.1007/s00125-020-05180-x

For peer review only

Figure legends

Figure 1. Flow chart of study population

Figure 2. Prevalence of prediabetes, unknown diabetes and known diabetes among 206 consecutive ICU patients with Covid-19

Figure 3. Probability of survival in no chronic dysglycemia patients and in patients with chronic dysglycemi

For peer review only

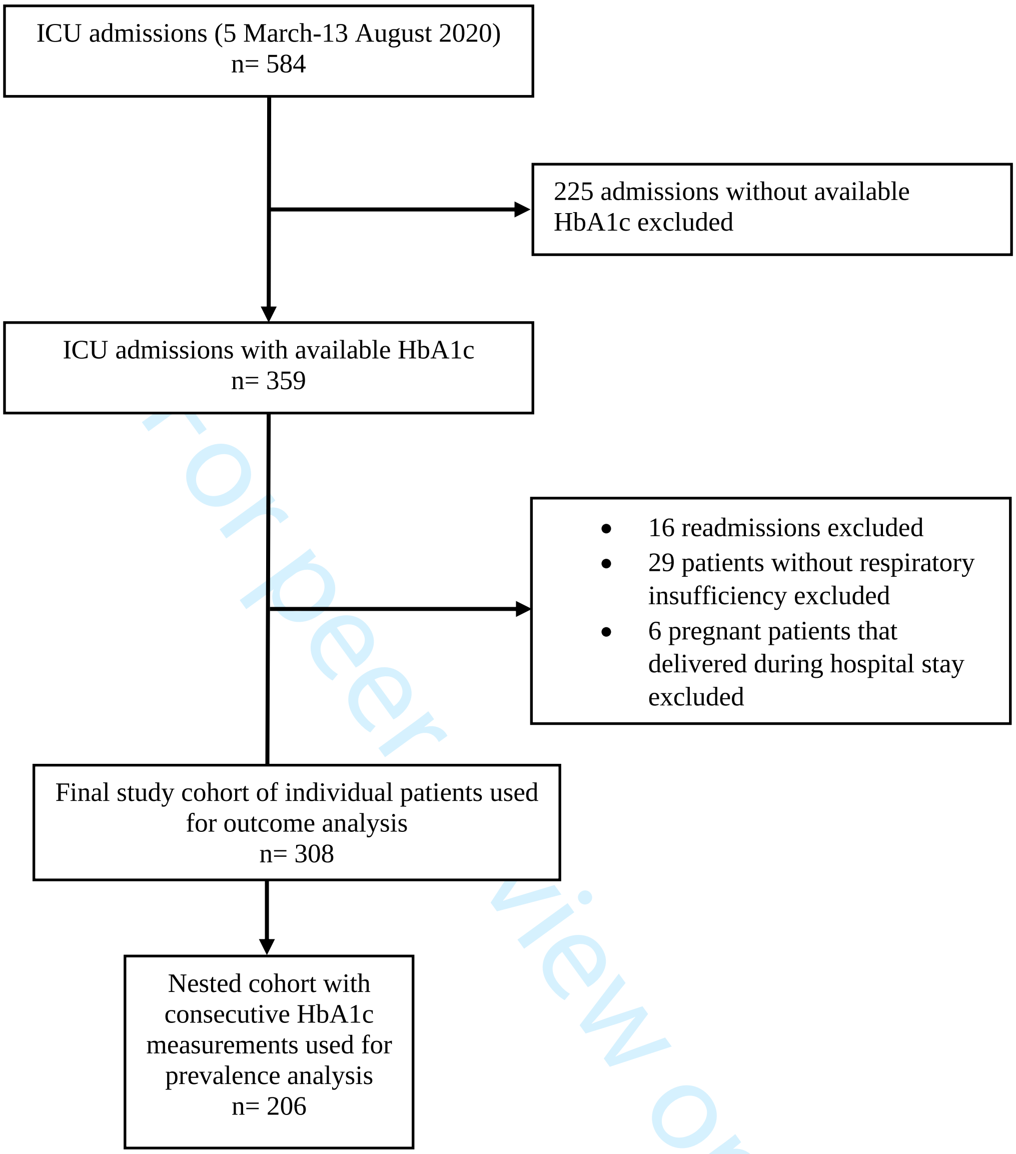

For peer review only

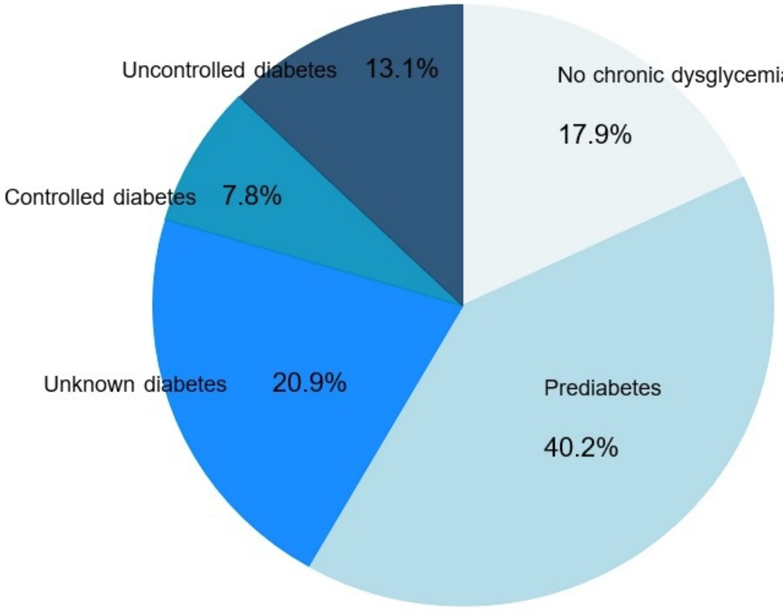

Figure 2. Prevalence of prediabetes, unknown diabetes and known diabetes among 206 consecutive ICU patients with Covid-19

144x102mm (144 x 144 DPI)

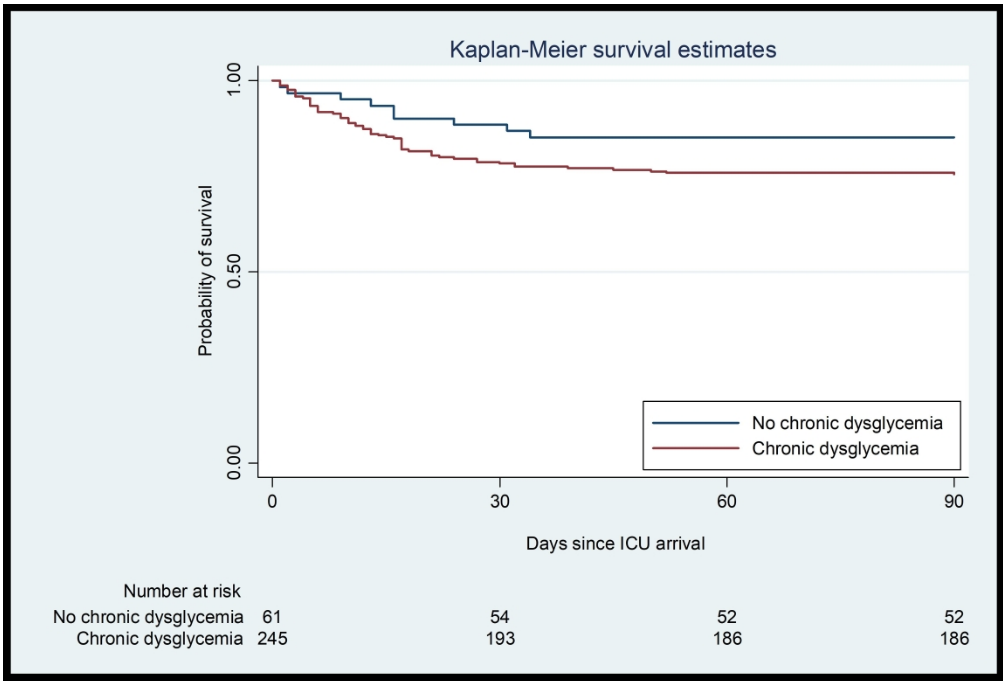

Figure 3. Probability of survival in no chronic dysglycemia patients and in patients with chronic dysglycemi

770x521mm (144 x 144 DPI)

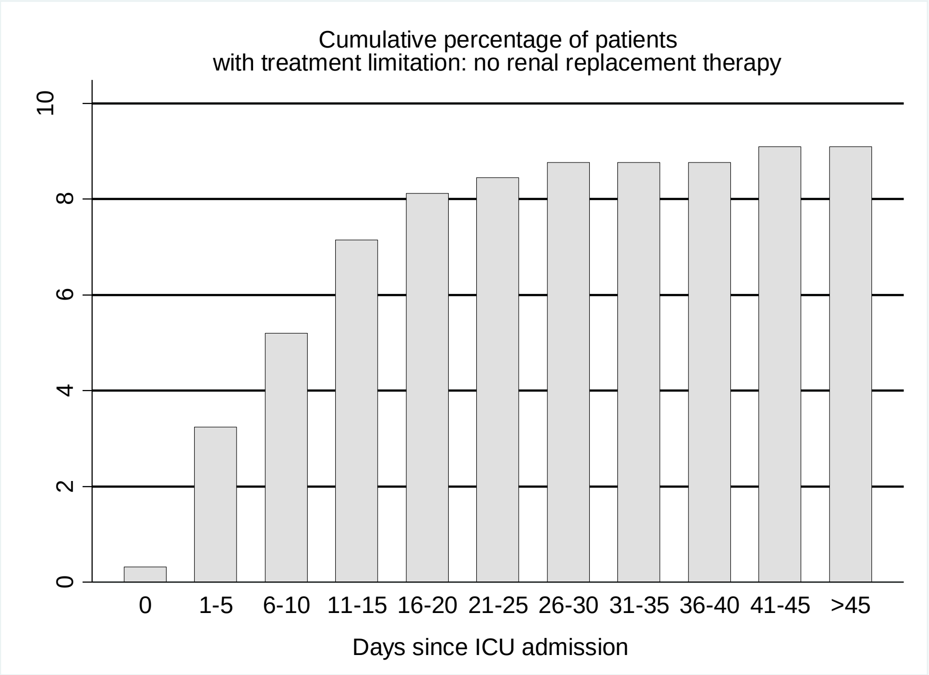

Figure S1. Cumulative percentage of patients with a treatment limitation: no renal replacement therapy. Day 0 (zero) denotes the day of admission to the intensive care unit.

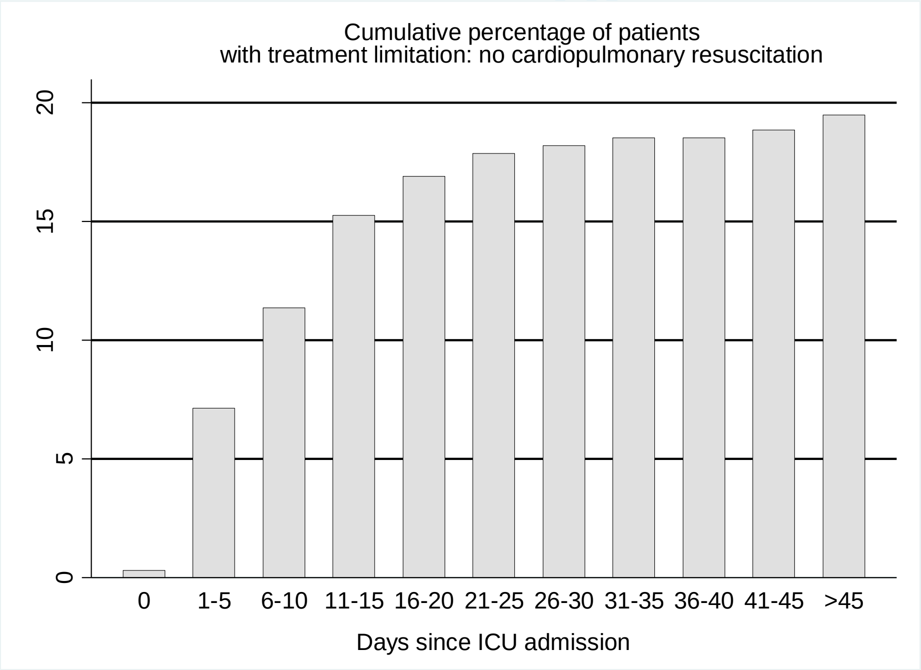

Figure S2. Cumulative percentage of patients with a treatment limitation: no cardiopulmonary resuscitation. Day 0 (zero) denotes the day of admission to the intensive care unit.

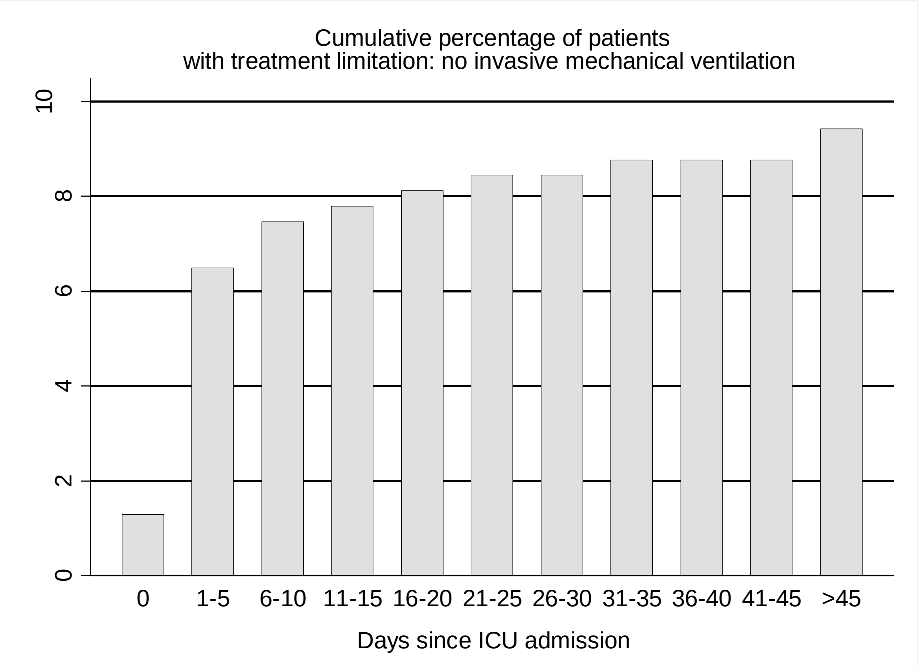

Figure S3. Cumulative percentage of patients with a treatment limitation: no invasive mechanical ventilation. Day 0 (zero) denotes the day of admission to the intensive care unit.

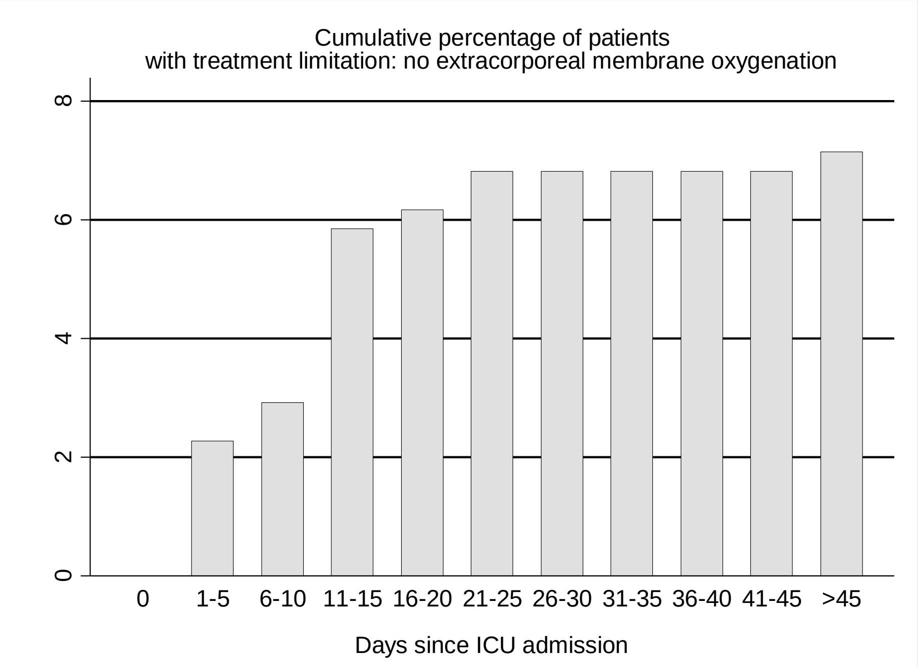

Figure S4. Cumulative percentage of patients with a treatment limitation: no extracorporeal membrane oxygenation. Day 0 (zero) denotes the day of admission to the intensive care unit.

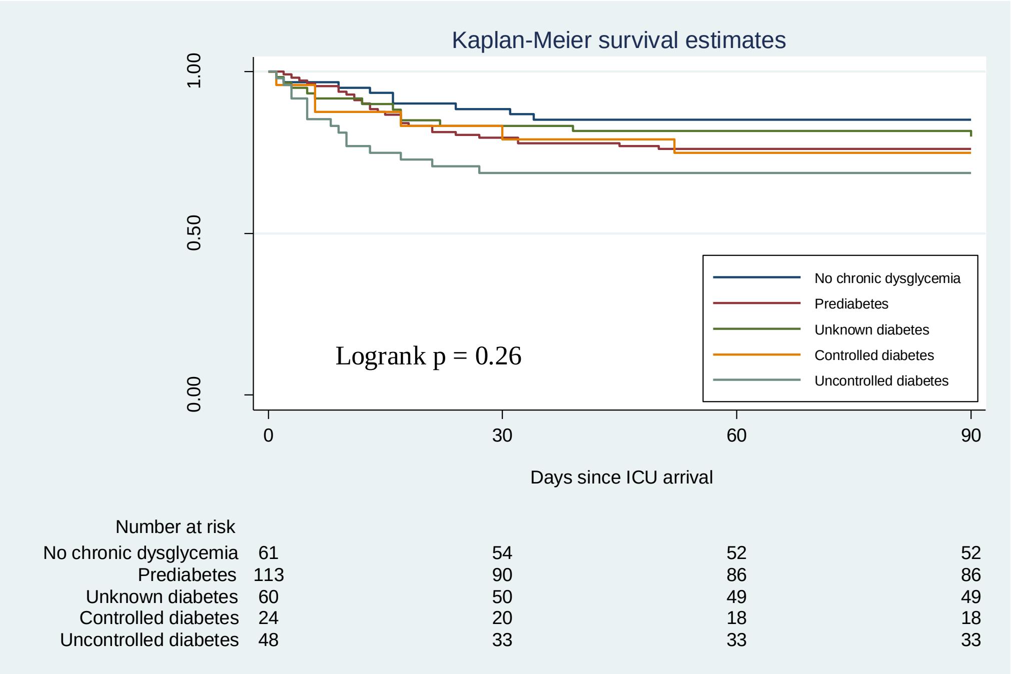

Figure S5. Probability of survival in the study groups

STROBE Statement—Checklist of items that should be included in reports of ***cohort studies***

Page references for document: **Chronic dysglycemia in critically ill patients with Covid-19 – a retrospective cohort study**

	Item No	Recommendation	Page No
**Title and abstract**	1	(*a*) Indicate the study's design with a commonly used term in the title or the abstract	1
		(*b*) Provide in the abstract an informative and balanced summary of what was done and what was found	2-3
**Introduction**			
Background/rationale	2	Explain the scientific background and rationale for the investigation being reported	3-4
Objectives	3	State specific objectives, including any prespecified hypotheses	4
**Methods4**			
Study design	4	Present key elements of study design early in the paper	4, 5
Setting	5	Describe the setting, locations, and relevant dates, including periods of recruitment, exposure, follow-up, and data collection	5-6
Participants	6	(*a*) Give the eligibility criteria, and the sources and methods of selection of participants. Describe methods of follow-up	5-7
		(*b*) For matched studies, give matching criteria and number of exposed and unexposed	NA
Variables	7	Clearly define all outcomes, exposures, predictors, potential confounders, and effect modifiers. Give diagnostic criteria, if applicable	7-8
Data sources/ measurement	8*	For each variable of interest, give sources of data and details of methods of assessment (measurement). Describe comparability of assessment methods if there is more than one group	5-7
Bias	9	Describe any efforts to address potential sources of bias	5
Study size	10	Explain how the study size was arrived at	5-6, Figure 1
Quantitative variables	11	Explain how quantitative variables were handled in the analyses. If applicable, describe which groupings were chosen and why	6-8
Statistical methods	12	(*a*) Describe all statistical methods, including those used to control for confounding	7
		(*b*) Describe any methods used to examine subgroups and interactions	7
		(*c*) Explain how missing data were addressed	8
		(*d*) If applicable, explain how loss to follow-up was addressed	NA
		(*e*) Describe any sensitivity analyses	NA
**Results**			
Participants	13*	(a) Report numbers of individuals at each stage of study—eg numbers potentially eligible, examined for eligibility, confirmed eligible, included in the study, completing follow-up, and analysed	8-9, Figure 1
		(b) Give reasons for non-participation at each stage	Figure 1
		(c) Consider use of a flow diagram	Figure 1

For peer review only

Descriptive data	14*	(a) Give characteristics of study participants (eg demographic, clinical, social) and information on exposures and potential confounders	8-9 Table 1
		(b) Indicate number of participants with missing data for each variable of interest	Table 1
		(c) Summarise follow-up time (eg, average and total amount)	NA
Outcome data	15*	Report numbers of outcome events or summary measures over time	10-11 Table 2-3

For peer review only

Main results	16	(*a*) Give unadjusted estimates and, if applicable, confounder-adjusted estimates and their precision (eg, 95% confidence interval). Make clear which confounders were adjusted for and why they were included<br>(*b*) Report category boundaries when continuous variables were categorized<br>(*c*) If relevant, consider translating estimates of relative risk into absolute risk for a meaningful time period	10-11 Table 3
Other analyses	17	Report other analyses done—eg analyses of subgroups and interactions, and sensitivity analyses	11 Figure 2,3,S1-4, S5
**Discussion**			
Key results	18	Summarise key results with reference to study objectives	11
Limitations	19	Discuss limitations of the study, taking into account sources of potential bias or imprecision. Discuss both direction and magnitude of any potential bias	14
Interpretation	20	Give a cautious overall interpretation of results considering objectives, limitations, multiplicity of analyses, results from similar studies, and other relevant evidence	15
Generalisability	21	Discuss the generalisability (external validity) of the study results	13-14
**Other information**			
Funding	22	Give the source of funding and the role of the funders for the present study and, if applicable, for the original study on which the present article is based	15

*Give information separately for exposed and unexposed groups.

Note: An Explanation and Elaboration article discusses each checklist item and gives methodological background and published examples of transparent reporting. The STROBE checklist is best used in conjunction with this article (freely available on the Web sites of PLoS Medicine at http://www.plosmedicine.org/, Annals of Internal Medicine at http://www.annals.org/, and Epidemiology at http://www.epidem.com/). Information on the STROBE Initiative is available at http://www.strobe-statement.org.

For peer review only

BMJ Open

Prevalence and impact of chronic dysglycemia among patients with Covid-19 in Swedish intensive care units: a multicenter, retrospective cohort study

Journal:	*BMJ Open*
Manuscript ID	bmjopen-2022-071330.R1
Article Type:	Original research
Date Submitted by the Author:	09-May-2023

Complete List of Authors:	Balintescu, Anca; Karolinska Institute, Department of Clinical Science and Education, Södersjukhuset<br>Rysz, Susanne; Karolinska Institute, Department of Perioperative Medicine and Intensive Care<br>Hertz, Carl; Stockholm South General Hospital Anaesthesia Department ICU<br>Grip, Jonathan; Karolinska Institute, Department of Perioperative Medicine and Intensive Care<br>Cronhjort, Maria; Karolinska Institute, Department of Clinical Science and Education Södersjukhuset<br>Oldner, Anders; Karolinska Institute, Department of Perioperative Medicine and Intensive Care<br>Svensen, Christer; Karolinska Institute, Department of Clinical Science and Education Södersjukhuset<br>Mårtensson, Johan; Karolinska Institute, Department of Perioperative Medicine and Intensive Care

**Primary Subject Heading**:	Intensive care
Secondary Subject Heading:	Diabetes and endocrinology
Keywords:	Adult intensive & critical care < INTENSIVE & CRITICAL CARE, COVID-19, DIABETES & ENDOCRINOLOGY, General diabetes < DIABETES & ENDOCRINOLOGY

SCHOLARONE™
Manuscripts

I, the Submitting Author has the right to grant and does grant on behalf of all authors of the Work (as defined in the below author licence), an exclusive licence and/or a non-exclusive licence for contributions from authors who are: i) UK Crown employees; ii) where BMJ has agreed a CC-BY licence shall apply, and/or iii) in accordance with the terms applicable for US Federal Government officers or employees acting as part of their official duties; on a worldwide, perpetual, irrevocable, royalty-free basis to BMJ Publishing Group Ltd ("BMJ") its licensees and where the relevant Journal is co-owned by BMJ to the co-owners of the Journal, to publish the Work in this journal and any other BMJ products and to exploit all rights, as set out in our licence.

The Submitting Author accepts and understands that any supply made under these terms is made by BMJ to the Submitting Author unless you are acting as an employee on behalf of your employer or a postgraduate student of an affiliated institution which is paying any applicable article publishing charge ("APC") for Open Access articles. Where the Submitting Author wishes to make the Work available on an Open Access basis (and intends to pay the relevant APC), the terms of reuse of such Open Access shall be governed by a Creative Commons licence – details of these licences and which Creative Commons licence will apply to this Work are set out in our licence referred to above.

Other than as permitted in any relevant BMJ Author's Self Archiving Policies, I confirm this Work has not been accepted for publication elsewhere, is not being considered for publication elsewhere and does not duplicate material already published. I confirm all authors consent to publication of this Work and authorise the granting of this licence.

For peer review only

Prevalence and impact of chronic dysglycemia among patients with Covid-19 in Swedish intensive care units: a multicenter, retrospective cohort study

Anca Balintescu, MD, PhD[1, 2]; Susanne Rysz, MD[3, 4]; Carl Hertz, MD[2]; Jonathan Grip, MD, PhD[3, 5]; Maria Cronhjort, MD, PhD[1, 2]; Anders Oldner MD, PhD[3, 6]; Christer Svensen MD, PhD[1, 2]; Johan Mårtensson, MD, PhD[3, 6]

[1] Department of Clinical Science and Education Karolinska Institute, Unit of Anesthesiology and Intensive Care, South General Hospital, Sjukhusbacken 10, 11883 Stockholm, Sweden

[2] Department of Anesthesia and Intensive Care, South General Hospital, Sjukhusbacken 10, 11883 Stockholm, Sweden

[3] Department of Perioperative Medicine and Intensive Care, Karolinska University Hospital, Norrbacka S2:05, SE-17176 Stockholm, Sweden

[4] Department of Medicine, Solna, Karolinska Institute, Stockholm, Sweden

[5] Department of Clinical Science, Intervention and Technology, Karolinska Institute, Stockholm, Sweden

[6] Department of Physiology and Pharmacology, Section of Anaesthesia and Intensive Care, Karolinska Institutet, Norrbacka S2:05, SE-17176 Stockholm, Sweden

Corresponding Author:

Anca Balintescu, ANOPIVA, South General Hospital, Sjukhusbacken 10, 11883 Stockholm, Sweden. Phone: +46 (0)722 7023 83. Fax: +46 (0)8-616 22 08. Email: anca.balintescu@ki.se

For peer review only - http://bmjopen.bmj.com/site/about/guidelines.xhtml

Keywords: HbA1c, ICU, Covid-19, prevalence, glycemic control, diabetes

Abstract

Objective: Using glycated hemoglobin A1c (HbA1c) screening, we aimed to determine the prevalence of chronic dysglycemia among patients with Covid-19 admitted to the intensive care unit (ICU). Additionally, we aimed to explore the association between chronic dysglycemia and clinical outcomes related to ICU stay.

Design: Multicenter retrospective observational study

Setting: ICUs in three hospitals in Stockholm, Sweden

Participants: Covid-19 patients admitted to the ICU between 5 March and 13 August 2020 with available HbA1c at admission. Chronic dysglycemia was determined based on previous diabetes history and HbA1c.

Primary and secondary outcomes: Primary outcome was the actual prevalence of chronic dysglycemia (prediabetes, unknown diabetes, or known diabetes) among Covid-19 patients. Secondary outcome was the association of chronic dysglycemia with 90-day mortality, ICU length of stay, duration of invasive mechanical ventilation (IMV), and renal replacement therapy (RRT), accounting for treatment selection bias.

Results: A total of 308 patients with available admission HbA1c were included. Chronic dysglycemia prevalence assessment was restricted to 206 patients admitted ICUs in which HbA1c was measured on all admitted patients. Chronic dysglycemia was present in 82.0% (95% CI 76.1%-87.0%) of patients, with prediabetes present in 40.2% (95% CI 33.5%-47.3%), unknown diabetes in 20.9% (95% CI 15.5%-27-1%), well-controlled diabetes in 7.8% (95% CI 4.5%-12.3%), and uncontrolled diabetes in 13.1% (95% CI 8.8%-18.5%). All patients with available HbA1c were included for the analysis of the relationship between chronic dysglycemia and secondary outcomes.

For peer review only - http://bmjopen.bmj.com/site/about/guidelines.xhtml

We found no independent association between chronic dysglycemia and 90-day mortality, ICU length of stay, or duration of IMV. After excluding patients with specific treatment limitations, no association between chronic dysglycemia and RRT use was observed.

Conclusions: In our cohort of critically ill Covid-19 patients, the prevalence of chronic dysglycemia was 82%. We found no robust associations between chronic dysglycemia and clinical outcomes when accounting for treatment limitations.

Strengths and limitations of this study

- Presents prevalence of chronic dysglycemia in an ICU population with Covid-19 based on additional quantification of admission HbA1c
- Actual prevalence of chronic dysglycemia calculation in all ICU admitted patients, reducing the risk of ascertainment bias
- Treatment limitations were considered in the analysis of clinical outcomes, thereby reducing the risk of treatment selection bias.
- We lack data on glycemic control during ICU stay, that might have influenced clinical outcomes

Background

Diabetes has been identified as a frequent comorbidity in patients with Covid-19, with a prevalence ranging from 7.4% to 34.3% among those requiring hospitalization [1-3]. A meta-analysis published in April 2020 found diabetes to be the second most frequent comorbidity in patients with Covid-19 admitted to the intensive care unit

For peer review only - http://bmjopen.bmj.com/site/about/guidelines.xhtml

(ICU) [4]. Furthermore, Covid-19 patients with diabetes appear to have a significantly higher risk of ICU admission and worse prognosis than Covid-19 patients without diabetes [4-6]. Particularly, a glycated hemoglobin A1c (HbA1c) level above 7% (53 mmol/mol) was identified as risk factor for ICU admission [7].

Recent data also indicates that diabetes is associated with worse prognosis among ICU patients with Covid-19 [8]. However, these studies did not include HbA1c measurements to identify patients with prediabetes or previously undiagnosed diabetes. This is an important limitation since both prediabetes and diabetes is considerably under-diagnosed both in the community [9] and in the ICU [10]. Additionally, a history of diabetes diagnosis and HbA1c at ICU admission, measured in consecutively admitted patients, is important in determining the true prevalence of chronic dysglycemia in the critically ill Covid-19 population. Finally, information about limitations of life-sustaining treatment were not considered in previous outcome analyses. This is unfortunate since the presence of such limitations may introduce treatment selection bias.

We therefore conducted a multicenter observational study using quantification of HbA1c and information about diabetes history to determine the actual prevalence of chronic dysglycemia (prediabetes, unknown diabetes, or known diabetes) among Covid-19 patients admitted to ICU. In addition, we aimed to explore the relationship of chronic dysglycemia with 90-day mortality, ICU length of stay, duration of invasive mechanical ventilation (IMV) and severe acute kidney injury requiring renal replacement therapy (RRT) accounting for treatment selection bias. We hypothesised that prevalence of chronic dysglycemia in Covid-19 patients admitted to the ICU exceeds the prevalence of chronic dysglycemia in the non Covid-19 critically ill

For peer review only - http://bmjopen.bmj.com/site/about/guidelines.xhtml

population. Moreover, we hypothesised that such chronic dysglycemia would be associated with worse clinical outcomes during ICU stay in patients with Covid-19.

Material and Methods

The study was approved by the Swedish Ethical Review Authority (approval number 2020-01302, amendment 2020-02890) with a waiver of informed consent. The study was performed in accordance with the Helsinki Declaration and reported in conformity with the STROBE statement [11]

Patient and Public Involvement statement: The study is based on data that was collected during the ongoing Covid-19 pandemic in a quality register. No intervention was applied to the individual patient. The public and patients were not involved in the design of the study. Results are to be disseminated to the public and scientific community through publication in peer-reviewed journal with open access.

Study design

We conducted a multicenter, retrospective observational study of adult (≥18 years) patients with a positive polymerase chain reaction (PCR) for severe acute respiratory syndrome coronavirus 2 (SARS-CoV-2) admitted to ten ICUs in three hospitals in Stockholm, Sweden between March 5th and August 13th, 2020 (first wave). We excluded patients without HbA1c obtained on admission to the ICU, patients in the third trimester of pregnancy and patients with a primary admission diagnosis other than Covid-19. In patients with multiple ICU admissions, only the first admission was considered. All included patients were assessed in the outcome analyses.

Assessment of chronic dysglycemia prevalence was restricted to a nested cohort of

For peer review only - http://bmjopen.bmj.com/site/about/guidelines.xhtml

patients from ICUs in which HbA1c measurement was included in the routine laboratory panel performed on all consecutive admissions. In the prevalence analysis, we therefore excluded patients with available HbA1c who were admitted to ICUs in which HbA1c was measured only at the discretion of the treating clinicians.

Data collection

HbA1c was measured in whole blood at ICU admission using the VARIANT II TURBO Hemoglobin Testing System analyzer (Bio-Rad Laboratories GmBH) and was reported in mmol/mol (IFCC calibrated) and in %. HbA1c was measured as part of routine care in three ICUs and at the discretion of the treating clinician in seven ICUs. We collected information on demographics, comorbidity, chronic medication, HbA1c value, mortality and decision regarding limitation of life-sustaining care from the patients' medical records (Take Care [CompuGroup Medical, Koblenz, Germany]). International Classification of Disease (ICD) 10 codes were used to identify comorbidity and previous history of diabetes. Additionally, data regarding known diabetes diagnosis was extracted manually from the patients' medical records. Data on body mass index (BMI), Simplified Acute Physiology Score (SAPS) 3, ICU length of stay, duration of IMV and RRT were collected from the ICU electronic patient data management system Clinisoft (GE, Barringgton, IL).

For peer review only

Prediabetes and Diabetes definitions

Prediabetes and diabetes were diagnosed based on two complementary methods; level of HbA1c at admission and previous medical history of diabetes, and categorized into five groups:

(1) no diabetes (HbA1c <42 mmol/mol [6.0%] and no history of diabetes)

For peer review only - http://bmjopen.bmj.com/site/about/guidelines.xhtml

(2) prediabetes (HbA1c 42-47 mmol/mol [6.0-6.4%] and no history of diabetes)

(3) unknown diabetes (HbA1c ≥48 mmol/mol [6.5 %] and no history of diabetes)

(4) controlled diabetes (HbA1c <52 mmol/mol [6.9 %] and previous history of diabetes)

(5) uncontrolled diabetes (HbA1c ≥52 mmol/mol [6.9 %] and previous history of diabetes).

Cut off values for HbA1c according to the World Health Organization were used [12]. Individuals in group (2), (3), (4) and (5) were considered to have chronic dysglycemia compared to those in group (1) labeled “no chronic dysglycemia”.

Outcomes

The primary outcome was the prevalence of chronic dysglycemia. Secondary outcomes included 90-day mortality, ICU length of stay, duration of IMV, and RRT use.

Statistical Analysis

We analyzed data using STATA version 12.1 (Stata Corp., College Station, Texas). Categorical data is presented as numbers and percentages and compared using the Fisher's exact test. Continuous data is summarized as median with interquartile range (IQR) and compared using the Mann-Whitney U test. The prevalence of chronic dysglycemia (primary outcome) was presented as percentages with 95% confidence intervals (CI). We displayed time to death within 90 days using Kaplan-Meier curves. Survival curves were compared using a log-rank test. We used multivariable Cox regression analysis to assess the association between chronic dysglycemia and 90-day mortality. We used multivariable linear regression analysis

For peer review only - http://bmjopen.bmj.com/site/about/guidelines.xhtml

to assess the association with ICU length of stay and duration of IMV. Both these outcomes were found to be well approximated by log-normal distributions and were therefore log-transformed before analysis with results presented as geometric means (95% CI). We used multivariable logistic regression analysis to assess the association with RRT use, before and after excluding patients with RRT as a treatment limitation. All regression analyses were conducted using the following models: adjusted for SAPS 3, age and sex, and adjusted for SAPS 3, age, sex, hypertension, any malignancy, any treatment limitation on admission and chronic corticosteroid use. A post-hoc exploratory comparison between subgroups was done for 90 day mortality and RRT use. A two-sided P-value <0.05 was considered statistically significant.

Results

Patients

A total of 584 patients with positive SARS-CoV-2 test were admitted to the study ICUs during the study period. We excluded 225 patients without available HbA1c, six pregnant patients, 16 readmissions and 29 patients without symptoms associated with Covid-19. Therefore, we included 308 patients with available HbA1c for outcome analysis. Among those 308 patients, 206 consequently admitted patients in which HbA1c was included in the admission routine laboratory panel were used for prevalence calculation (Figure 1). Baseline characteristics and treatment limitations of the entire study population are detailed in Table 1.

For peer review only - http://bmjopen.bmj.com/site/about/guidelines.xhtml

Table 1. Baseline characteristics and treatment limitations

		Chronic dysglycemia				
**Characteristic**	**No chronic dysglycemia**	**Prediabetes**	**Unknown diabetes**	**Controlled diabetes**	**Uncontrolled diabetes**	**P[e]**
No. (%)	61 (19.8)	114 (37.0)	60 (19.4)	25 (8.11)	48 (15.5)	
Age, years	57 (51,63)	61 (53, 68)	60 (52, 68)	63 (57, 71)	62 (55,69)	0.03
Male sex	48 (78.6)	92 (80.7)	47 (78.3)	21 (84.0)	36 (75.0)	1.00
Body mass index[a], kg/m^2	27 (25, 32)	27 (25, 30)	28 (25, 31)	29 (26, 32)	30 (26, 33)	0.97
HbA1c, mmol/mol	39 (36, 40)	44 (43, 46)	51 (49, 57)	47 (44, 49)	70 (61, 81)	<0.001
Diabetes treatment						
Diet only				6 (24.0)	2 (4.1)	
OAD only				17 (68)	19 (39.5)	
Insulin only				1 (4.0)	12 (25.0)	
OAD+Insulin				1 (4.0)	15 (31.2)	
Comorbidity						
Hypertension	18 (29.5)	40 (35.0)	23 (38.3)	16 (64.0)	34 (70.8)	0.02
Heart failure	6 (9.8)	5 (4.3)	6 (10.0)	0 (0.0)	3 (6.2)	0.24
Previous myocardial infarction	2 (3.2)	4 (3.5)	6 (10.0)	0 (0.0)	7 (14.5)	0.38
Chronic kidney disease	4 (6.5)	13 (11.4)	7 (11.6)	6 (24.0)	11 (22.9)	0.09
Liver disease	2 (3.2)	4 (3.5)	1 (1.6)	1 (4.0)	1 (2.0)	1.00
Any malignancy	0 (0.0)	8 (7.0)	2 (3.3)	2 (8.0)	4 (8.3)	0.04
Astma/COPD	13 (21.3)	20 (17.5)	14 (23.3)	5 (20.0)	9 (18.7)	0.72
SAPS 3[b]	53 (48, 60)	55 (49, 60)	57 (52, 62)	59 (52, 63)	56 (52, 69)	0.18
Chronic drug use						
Corticosteroids[c]	5 (8.20)	16 (14.04)	8 (13.3)	4 (16.0)	6 (12.5)	0.24
Immunosuppressive therapy[d]	1 (1.6)	8 (7.0)	3 (5.0)	1 (4.0)	1(2.0)	0.31
Treatment limitations[f]						
Any limitation	14 (22.9)	19 (16.6)	13 (21.6)	8 (32.0)	13 (27.0)	0.86
No RRT	5 (8.2)	7 (6.1)	5 (8.3)	5 (20.0)	6 (12.5)	1.00
No IMV	6 (9.8)	10 (8.7)	4 (6.6)	4 (16.0)	6 (12.5)	1,00
No CPR	9 (14.7)	19 (16.6)	12 (20.0)	8 (32.0)	12 (25.0)	0.36
No ECMO	7 (11.4)	3 (2.6)	5 (8.3)	3 (12.0)	3 (6.2)	0.15
Palliative care[g]	1 (1.6)	18 (16.5)	7 (12.2)	4 (16.6)	5 (10.8)	0.006

Data are n (%) or median (interquartile range)
HbA1c, glycated hemoglobin A1c; OAD, oral hypoglycemic agent; COPD, Chronic obstructive pulmonary disease; SAPS, Simplified Acute Physiology Score, 2 missing values (306 patients with data); RRT, renal replacement therapy; IMV, invasive mechanical ventilation; CPR, cardiopulmonary resuscitation
[a]Missing data in 15 patients (293 patients with data)
[b]Missing data in 2 patients (306 patients with data)
[c]Systemic or inhalatory corticosteroids
[d]Immunosuppressive therapy was defined as: treatment with Metotrexate, Azatioprin, Ciklosporin, Tracolimus, Infliximab
[e]P values for the comparison between no chronic dysglycemia and chronic dysglycemia, Mann-Whitney U test was used for comparison of continuous data and Fischer's exact test for comparison of categorical data
[f] Decision taken any time during ICU stay
[g]Decision to go over to palliative care taken during ICU stay

Patients with chronic dysglycemia were older, were more likely to have hypertension, malignancy and/or chronic kidney disease, and had higher SAPS 3 than patients without chronic dysglycemia. Overall, 14 (22.9%) patients in the no chronic dysglycemia group and 53 (21.5%) patients in the chronic dysglycemia group

For peer review only - http://bmjopen.bmj.com/site/about/guidelines.xhtml

received one or more limitations of life-supporting therapies during their ICU stay. “No Cardiopulmonary resuscitation (CPR)” was the most common treatment limitation. We observed the highest proportion of limitations among patients with known (controlled or uncontrolled) diabetes. Decision to switch to palliative care was made in 1 (1.6%) patient in the no chronic dysglycemia group and 34 (13.8%) patients in the chronic dysglycemia group (P=0.006). Cumulative percentage of treatment limitations relative ICU admission is displayed in Figures S1-S4.

Primary outcome

In the nested cohort of 206 consecutive patients with available HbA1c, 169 (82.0%; 95% CI 76.1%-87.0%) were diagnosed with chronic dysglycemia. Prediabetes was present in 83 (40.2%, 95% CI 33.5%-47.3%), unknown diabetes in 43 (20.9%, 95% CI 15.5%-27-1%), well-controlled diabetes in 16 (7.8%, 95% CI 4.5%-12.3%), and uncontrolled diabetes in 27 (13.1%, 95% CI 8.8%-18.5%) patients (Figure 2).

Secondary outcomes

Nine (14.7%) patients in the no chronic dysglycemia group and 62 (25.1%) patients in the chronic dysglycemia group died within 90 days (P=0.09) (Table 2, Figure 3 and Figure S5). ICU length of stay and duration of IMV were similar in the two groups. IMV was delivered to 42 (68.8%) patients without chronic dysglycemia and to 187 (75.7%) patients with chronic dysglycemia (P=0.32). RRT was delivered to 17 (27.9%) no chronic dysglycemia patients and 42 (17.0%) chronic dysglycemia patients (P=0.06) (Table 2 and Table 3).

For peer review only - http://bmjopen.bmj.com/site/about/guidelines.xhtml

Table 2. Secondary outcomes

		Chronic dysglycemia				
**Outcomes**	**No chronic dysglycemia (n = 61)**	**Prediabetes (n = 114)**	**Unknown diabetes (n =60)**	**Controlled diabetes (n = 25)**	**Uncontrolled diabetes (n = 48)**	**P[a]**
90-day mortality, n (%)	9 (14.7)	28 (24.5)	12 (20.0)	7 (28.0)	15 (31.2)	0.09
ICU length of stay, days	9 (4, 25)	14 (6, 24)	13 (6, 28)	8 (5, 21)	11 (7, 22)	0.69
Invasive mechanical ventilation, days	16 (8, 29)	14 (10, 23)	15 (10, 27)	15 (6, 21)	14 (9, 22)	0.60
Renal replacement therapy, n (%)	17 (27.9)	22 (19.3)	11 (18.3)	1 (4.0)	8 (16.6)	0.06

Data are n (%) or median (interquartile range)
ICU lengths of stay (4 missing values because of transfer to other hospital)
[a] P values for the comparison between no chronic dysglycemia and chronic dysglycemia, Mann-Whitney U test was used for comparison of continuous data and Fischer's exact test for comparison of categorical data.

For peer review only

Table 3. Multivariable regression analyses showing the association of chronic dysglycemia (versus no chronic dysglycemia) with secondary outcomes

**Outcome measure**	**No Chronic Dysglycemia**	**Chronic Dysglycemia**	**Adjusted Risk Estimate (95% CI)[a]**	**P[a]**	**Adjusted Risk Estimate (95% CI)[b]**	**P[b]**	**Statistical test**
**90-day mortality n (%)**	9/61 (14.7)	62/247 (25.1)	1.61 (0.79 to 3.26)	0.18	1.54 (0.74 to 3.19)	0.24	Cox regression
**ICU length of stay, days**							
All patients	9 (4, 25)	13 (6, 23)	1.06 (0.78 to 1.43)	0.70	1.05 (0.77 to 1.44)	0.71	Linear regression
ICU survivors[c]	9 (5, 27)	14 (7, 24)	1.04 (0.76 to 1.43)	0.75	1.05 (0.76 to 1.44)	0.75	Linear regression
**Invasive mechanical ventilation duration, days**							
All patients[d]	16 (8, 29)	14 (10, 23)	0.92 (0.68 to 1.23)	0.58	0.93 (0.69 to 1.24)	0.63	Linear regression
ICU survivors[e]	16 (8, 30)	15 (10, 23)	0.93 (0.70 to 1.23)	0.61	0.92 (0.70 to 1.22)	0.59	Linear regression
**Renal replacement therapy, n (%)**							
All patients	17/61 (27.9)	42/247 (17.0)	0.52 (0.26 to 1.02)	0.06	0.49 (0.24 to 0.99)	0.04	Logistic regression
Patients without treatment limitation as no RRT	17/57 (29.8)	42/224 (18.8)	0.52 (0.26 to 1.04)	0.10	0.52 (0.25 to 1.07)	0.08	Logistic regression

[a]Multivariable models were adjusted for Simplified Acute Physiology Score (SAPS) 3, age and sex.
[b] Multivariable models were adjusted for Simplified Acute Physiology Score (SAPS) 3, age, sex, hypertension, any malignancy, any treatment limitation on admission and chronic corticosteroid use
[c]ICU length of stay in ICU survivors, 260 observations
[d]Invasive mechanical ventilation duration, 227 observations
[e]Invasive mechanical ventilation duration in ICU survivors, 189 observations

On multivariable regression analysis we observed a numerically higher mortality (adjusted HR 1.54, 95% CI 0.74-3.19, P=0.24) and significantly lower RRT use (adjusted OR 0.49, 95% CI 0.24-0.99, P=0.04) in patients with chronic dysglycemia (Table 3). No association with RRT was observed after exclusion of patients with "No RRT" as treatment limitation. In the post-hoc exploratory comparison between subgroups, RRT use was higher in the no diabetes group compared to the controlled diabetes group, as well as in the uncontrolled diabetes compared to controlled diabetes group (Table S1). Individuals with uncontrolled diabetes had the lowest probability of survival followed by individuals with controlled diabetes and prediabetes. The highest probability of survival was observed among patients with no chronic dysglycemia and prediabetes, respectively (Figure S5). However, we observed no statistically significant differences in mortality in the post-hoc comparison of subgroups (Table S1).

Discussions

Key findings

We performed a multicenter observational investigation to determine the prevalence of chronic dysglycemia and its impact on clinical outcomes among Covid-19 patients admitted to ICU. Using available information about the patients' diabetic status in combination with routine HbA1c assessment, we found that 82% had chronic dysglycemia with two thirds having either prediabetes or undiagnosed diabetes. We observed numerically higher 90-day mortality in patients with chronic dysglycemia, with the highest mortality (31%) observed among those with uncontrolled diabetes.

For peer review only - http://bmjopen.bmj.com/site/about/guidelines.xhtml

Conversely, the proportion of patients receiving RRT was lower among patients with chronic dysglycemia even when patients without "No RRT" as treatment limitation were considered separately. We found no association of chronic dysglycemia with ICU length of stay or duration of IMV.

Relationship with previous studies

A global meta-analysis of more than 16000 ICU patients with Covid-19 suggests a pooled prevalence of known diabetes between 23-31% [13], close to the observed prevalence in our study (21%). However, few studies have used additional HbA1c measurements to assess the actual prevalence of chronic dysglycemia, including prediabetes and undiagnosed diabetes. One such ICU study from Austria found a prevalence of chronic dysglycemia of 85%, which is in close agreement with our findings [14]. However, the Austrian study did not assess consecutive patients and may therefore be prone to selection bias.

Our findings indicate that chronic dysglycemia is more common in Covid-19 patients than in ICU patients with other admission diagnoses. In fact, in a pre-Covid-19 cohort of general ICU patients we found a corresponding dysglycemia prevalence of 33% [10]. The relationship between severe SARS-CoV-2 infection and dysglycemia has different potential explanations. SARS-CoV-2 enters cells in various organs, including the pancreas, via angiotensin-converting enzyme II (ACE2). As ACE2 is involved in regulating pancreatic beta-cell function, a link between SARS-CoV-2 infection and beta-cell dysfunction and diabetes development has been suggested [15].

Interestingly, the prevalence of elevated HbA1c below the diabetes diagnostic threshold (prediabetes) was markedly higher in our Covid-19 cohort than in our previous pre-Covid-19 cohort (40% vs 9%). It is possible that the duration of Covid-

For peer review only - http://bmjopen.bmj.com/site/about/guidelines.xhtml

19 symptoms before ICU admission (typically ten days in the literature [16]) was sufficient to trigger new onset hyperglycemia with mildly elevated HbA1c.

In addition to the above speculations about SARS-CoV-2 as a *cause* of dysglycemia, there is also evidence suggesting that patients with preexisting dysglycemia are prone to a more severe course of Covid-19. For example, some studies have shown that hospitalized SARS-CoV-2 positive with prediabetes, unknown diabetes, and known poorly controlled diabetes are at increased risk of SARS-CoV-2 associated respiratory failure requiring intensive care [17]. A higher burden of comorbidities, hyperglycemia *per se*, and chronic low-grade inflammation in diabetes may explain this observation [18].

Wang et al identifies fasting glucose as an independent predictor for 28-day mortality in hospitalized individuals with Covid-19 and previously unknown diabetes. However, HbA1c was not assessed and interference from stress hyperglycemia might have led to the different results compared to our study [19].

Others [20], identified an increased risk of death in individuals with diabetes and increasing levels of HbA1c above 48 mmol/mol and known diabetes in a large cohort of hospitalized patients, but not in critically ill individuals.

Whether chronic dysglycemia is associated with worse outcomes among Covid-19 patients admitted to ICU remains uncertain. Dennis et al [21] found increased mortality risk at 30 days (HR 1.23 [95% CI 1.14, 1.32]) compared to patients with no diabetes in patients admitted to the high Dependency Unit or ICU, but did not take HbA1c into consideration. A multicenter study from France including 410 ICU patients with Covid-19, found no association between the severity of dysglycemia and tracheal intubation and/or death within 7 days of admission in patients with diabetes than in those without diabetes [22]. This is in accordance with the findings of

For peer review only - http://bmjopen.bmj.com/site/about/guidelines.xhtml

our study. In contrast, others found higher mortality in the subgroup of mechanically ventilated patients with diabetes [14].

We previously demonstrated an independent association between chronic dysglycemia and need for RRT in critically ill non-Covid-19 patients [10]. This association was, however, not found in the present study. In fact, we observed a higher proportion of patients requiring RRT among our patients without chronic dysglycemia and an inverse association between chronic dysglycemia and RRT use. Only one individual in the controlled diabetes subgroup received RRT during ICU stay. We believe this surprising finding may be due to treatment limitations. In fact, after exclusion of patients with treatment limitation “not for RRT”, we observed no statistically significant association between chronic dysglycemia and RRT use. Limitations in life-sustaining care were more common in the known diabetes groups (well controlled and uncontrolled diabetes) than in all other groups. We cannot exclude the possibility that patients with severe acute or chronic kidney injury did not reach the ICU because of treatment limitation decisions made at hospital arrival or on the medical ward. This might have influenced the number of patients with kidney injury reaching the ICU, affecting predominantly patients with chronic dysglycemia, as they are usually older and have multiple comorbidities.

Strengths and limitations

Our study has several strengths. It is the first to assess the prevalence of chronic dysglycemia in an ICU population with Covid-19 based on additional quantification of admission HbA1c. This approach reduced bias due to events that would have influenced HbA1c values obtained before ICU admission. We restricted the prevalence assessment to a cohort of patients who were admitted to ICUs where

For peer review only - http://bmjopen.bmj.com/site/about/guidelines.xhtml

HbA1c was part of the routine laboratory panel, thereby reducing the risk of ascertainment bias. Additionally, by measuring HbA1c in all patients admitted to the ICU we identified 169 (82%) individuals with chronic dysglycemia and 86 (41.7%) with diabetes. If HbA1c would not have been measured routinely at ICU admission, we would only have identified 43 (20.9%) individuals with diabetes. Furthermore, we considered treatment limitations in our analysis of clinical outcomes, thereby reducing the risk of treatment selection bias. Finally, we included patients admitted to ten ICUs in three University hospitals, thus providing a degree of external validity for applying our findings to similar settings.

Our study has limitations. We lack data on conditions and treatment that might have influenced admission HbA1c, such as haemoglobinopathies and blood transfusion before ICU admission. Since interviews with patients or relatives were not performed, a degree of misclassification due to non-documented dysglycemia diagnoses cannot be ruled out. However, such interviews would have been logistically difficult during the ongoing pandemic. We used an HbA1c cutoff of 42-47 mmol/mol (6.0-6.4%) to classify prediabetes. If we instead had used the cutoff suggested by the American Diabetes Association (39-47 mmol/mol [5.7-6.4%], our prevalence of chronic dysglycemia would have increased from 82.0% to 91.3%. This approach did not, however, alter the association with the secondary outcomes (data not shown). In addition, we lack information about glycemic control during intensive care, which might have modified clinical outcomes.

The observational nature of the study does not imply causation. Generalizability of our results is limited to populations with similar health care systems and similar legal frame-works for decisions on treatment limitations. Finally, the limited sample size

For peer review only

may limit the conclusion regarding secondary outcomes that can be drawn from the data.

Conclusion

In our multicenter cohort of Covid-19 patients admitted to the ICU, HbA1c screening diagnosed chronic dysglycemia in four out of five patients with the majority having either prediabetes or previously undiagnosed diabetes. Chronic dysglycemia was not significantly associated with mortality, ICU length of stay, duration of invasive mechanical ventilation or renal replacement therapy use after considering treatment limitations. These findings indicate that chronic dysglycemia may be a risk factor for severe Covid-19. However, Covid-19 prognosis in the ICU does not appear to be modified by chronic dysglycemia.

Author Contributions:

AB, SR, MC, JG, AO, CS and JM contributed to the concept and design of the study.

AB, SR, CH and JM collected data.

AB, SR, CH, JG, MC, CS, AO and JM contributed to the analysis and interpretation of data.

AB and JM drafted the manuscript.

All authors critical reviewed and approved the final manuscript.

AB accepts full responsibility for the work and the conduct of the study, had access to the data, and controlled the decision to publish.

For peer review only - http://bmjopen.bmj.com/site/about/guidelines.xhtml

For peer review only

Funding AB and JM were supported by Region Stockholm (clinical research appointment and ALF project grants), grant number 580282.

A non-peer-reviewed version of this article has been a part of a publicly defended PhD thesis ISBN 978-91-8016-719-2 and was submitted to Karolinska Institute Open Archive, available at https://openarchive.ki.se/xmlui/handle/10616/48203 on 19 September 2022.

Competing interest: The authors have no competing interest relevant to this work.

Data availability: The data that support the findings of this study are available from the corresponding author upon reasonable request.

Ethics Approval Statement: The study was approved by the Swedish Ethical Review Authority (approval number 2020-01302).

References

[1] Yang J, Zheng Y, Gou X, Pu K, Chen Z, Guo Q, et al. Prevalence of comorbidities and its effects in patients infected with SARS-CoV-2: a systematic review and meta-analysis. International Journal of Infectious Diseases 2020;94:91-5. doi:10.1016/j.ijid.2020.03.017

[2] Liu Y, Lu R, Wang J, Cheng Q, Zhang R, Zhang S, et al. Diabetes, even newly defined by HbA1c testing, is associated with an increased risk of in-hospital death in adults with COVID-19. BMC Endocrine Disorders 2021;21(1):1-10. doi:10.1186/s12902-021-00717-6

[3] Guan W-j, Liang W-h, Zhao Y, Liang H-r, Chen Z-s, Li Y-m, et al. Comorbidity and its impact on 1590 patients with COVID-19 in China: a nationwide analysis. European Respiratory Journal 2020;55(5):2000547. doi:10.1183/13993003.00547-2020

[4] Roncon L, Zuin M, Rigatelli G, Zuliani G. Diabetic patients with COVID-19 infection are at higher risk of ICU admission and poor short-term outcome. J Clin Virol 2020;127:104354. doi:10.1016/j.jcv.2020.104354

[5] Zhou F, Yu T, Du R, Fan G, Liu Y, Liu Z, et al. Clinical course and risk factors for mortality of adult inpatients with COVID-19 in Wuhan, China: a retrospective cohort study. The Lancet 2020;395. doi:10.1016/S0140-6736(20)30566-3

[6] Guo W, Li M, Dong Y, Zhou H, Zhang Z, Tian C, et al. Diabetes is a risk factor for the progression and prognosis of COVID-19. Diabetes/metabolism research and reviews 2020:e3319.

For peer review only - http://bmjopen.bmj.com/site/about/guidelines.xhtml

[7] Lei M, Lin K, Pi Y, Huang X, Fan L, Huang J, et al. Clinical Features and Risk Factors of ICU Admission for COVID-19 Patients with Diabetes. Journal of Diabetes Research 2020;2020:5237840. doi:10.1155/2020/5237840

[8] Grasselli G, Greco M, Zanella A, Albano G, Antonelli M, Bellani G, et al. Risk Factors Associated With Mortality Among Patients With COVID-19 in Intensive Care Units in Lombardy, Italy. JAMA internal medicine 2020;180(10):1345-55. doi:10.1001/jamainternmed.2020.3539

[9] Beagley J, Guariguata L, Weil C, Motala AA. Global estimates of undiagnosed diabetes in adults. Diabetes Research and Clinical Practice 2014;103(2):150-60. doi:https://doi.org/10.1016/j.diabres.2013.11.001

[10] Balintescu A, Palmgren I, Lipcsey M, Oldner A, Larsson A, Cronhjort M, et al. Prevalence and impact of chronic dysglycemia in intensive care unit patients-A retrospective cohort study. Acta Anaesthesiol Scand 2021;65(1):82-91. doi:10.1111/aas.13695

[11] Erik von Elm DGA, Matthias Egger, Stuart J. Pocock, Peter C. Gøtzsche, and Jan P. Vandenbroucke. The Strengthening the Reporting of Observational Studies in Epidemiology (STROBE) Statement: Guidelines for Reporting Observational Studies. Annals of Internal Medicine 2007;147(8):573-7. doi:10.7326/0003-4819-147-8-200710160-00010 %m 17938396

[12] Organisation WH. World Health Organisation. Use of Glycated Haemoglobin (HbA1c) in the Diagnosis of Diabetes Mellitus, https://www.who.int/publications/i/item/use-of-glycated-haemoglobin-(-hba1c)-in-diagnosis-of-diabetes-mellitus; [accessed 07 Fenruary 2022].

[13] Tan E, Song J, Deane AM, Plummer MP. Global Impact of Coronavirus Disease 2019 Infection Requiring Admission to the ICU: A Systematic Review and Meta-analysis. Chest 2021;159(2):524-36. doi:https://doi.org/10.1016/j.chest.2020.10.014

[14] Klein SJ, Mayerhöfer T, Fries D, Preuß Hernández C, Joannidis M, Collaborators, et al. Elevated HbA1c remains a predominant finding in severe COVID-19 and may be associated with increased mortality in patients requiring mechanical ventilation. Critical Care 2021;25(1):1-4. doi:10.1186/s13054-021-03730-2

[15] Memon B, Abdelalim EM. ACE2 function in the pancreatic islet: Implications for relationship between SARS-CoV-2 and diabetes. Acta Physiologica 2021;233(4):1-13. doi:10.1111/apha.13733

[16] Larsson E, Brattstrom O, Agvald-Ohman C, Grip J, Jalde FC, Stralin K, et al. Characteristics and outcomes of patients with COVID-19 admitted to ICU in a tertiary hospital in Stockholm, Sweden. Acta anaesthesiologica Scandinavica 2021;65(1):76-81. doi:10.1111/aas.13694

[17] Rysz S, Jonsson Fagerlund M, Rimes-Stigare C, Larsson E, Campoccia Jalde F, Mårtensson J. Chronic dysglycemia and risk of SARS-CoV-2 associated respiratory failure in hospitalized patients. Acta anaesthesiologica Scandinavica 2022;66(1):48-55. doi:10.1136/bjsports-2021-104080.

[18] Landstra CP, de Koning EJP. COVID-19 and Diabetes: Understanding the Interrelationship and Risks for a Severe Course. Frontiers in endocrinology 2021;12:649525. doi:10.3389/fendo.2021.649525

[19] Wang S, Ma P, Zhang S, Song S, Wang Z, Ma Y, et al. Fasting blood glucose at admission is an independent predictor for 28-day mortality in patients with COVID-19 without previous diagnosis of diabetes: a multi-centre retrospective study. Diabetologia 2020;63(10):2102-11. doi:10.1007/s00125-020-05209-1

[20] Holman N, Knighton P, Kar P, O'Keefe J, Curley M, Weaver A, et al. Risk factors for COVID-19-related mortality in people with type 1 and type 2 diabetes in England: a

For peer review only - http://bmjopen.bmj.com/site/about/guidelines.xhtml

population-based cohort study. The lancet Diabetes & endocrinology 2020;8(10):823-33. doi:10.1016/S2213-8587(20)30271-0
[21] Dennis JM, Mateen BA, Sonabend R, Thomas NJ, Patel KA, Hattersley AT, et al. Type 2 Diabetes and COVID-19-Related Mortality in the Critical Care Setting: A National Cohort Study in England, March-July 2020. Diabetes Care 2021;44(1):50-7. doi:10.2337/dc20-1444
[22] Cariou B, Hadjadj S, Wargny M, Pichelin M, Al-Salameh A, Allix I, et al. Phenotypic characteristics and prognosis of inpatients with COVID-19 and diabetes: the CORONADO study. Diabetologia 2020;63(8):1500-15. doi:10.1007/s00125-020-05180-x

Figure legends

Figure 1. Flow chart of study population

Figure 2. Prevalence of prediabetes, unknown diabetes and known diabetes among 206 consecutive ICU patients with Covid-19

Figure 3. Probability of survival in no chronic dysglycemia patients and in patients with chronic dysglycemia

For peer review only

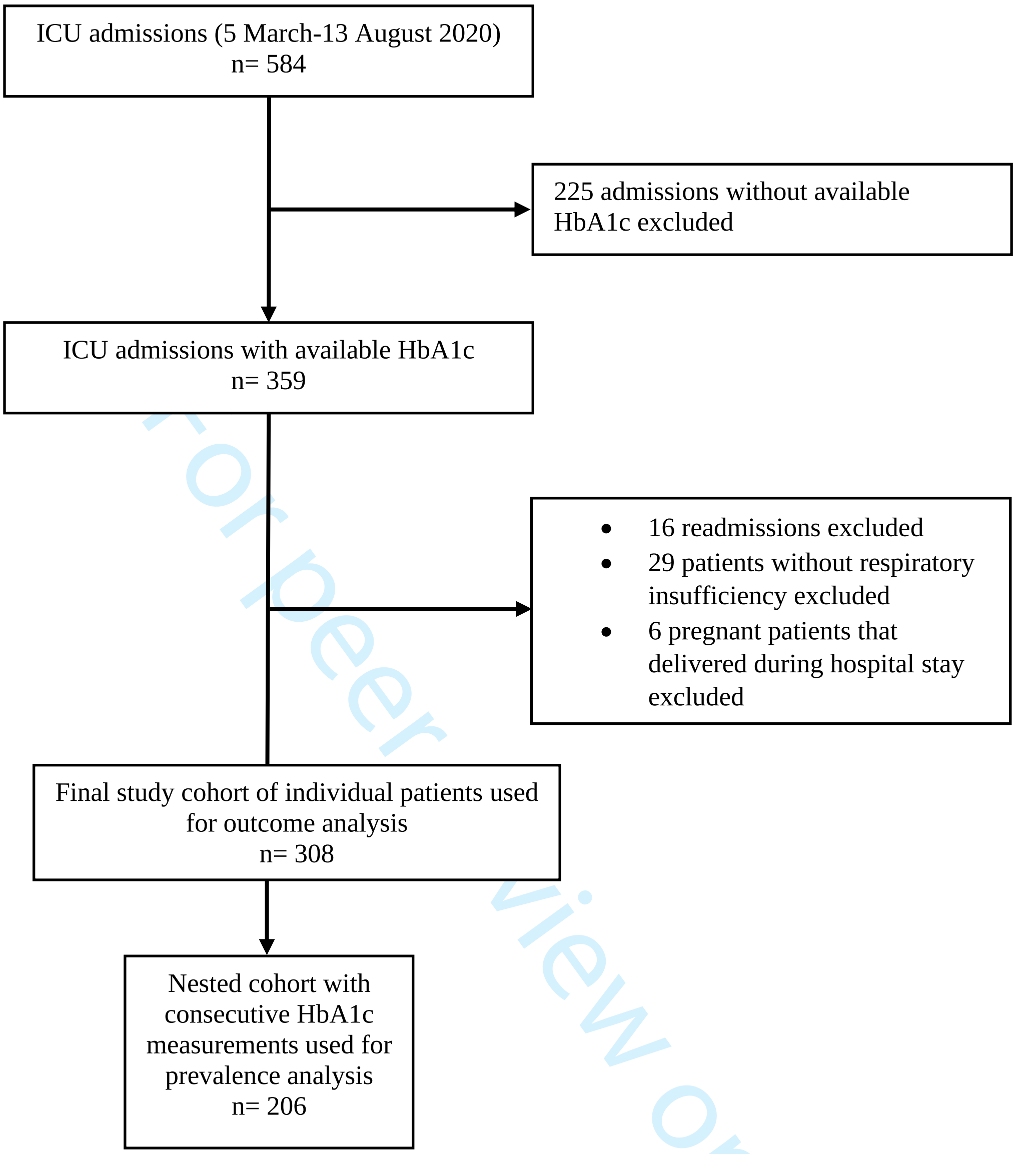
ICU admissions (5 March-13 August 2020)
n= 584
225 admissions without available HbA1c excluded
ICU admissions with available HbA1c
n= 359
16 readmissions excluded
29 patients without respiratory insufficiency excluded
6 pregnant patients that delivered during hospital stay excluded
Final study cohort of individual patients used for outcome analysis
n= 308
Nested cohort with consecutive HbA1c measurements used for prevalence analysis
n= 206

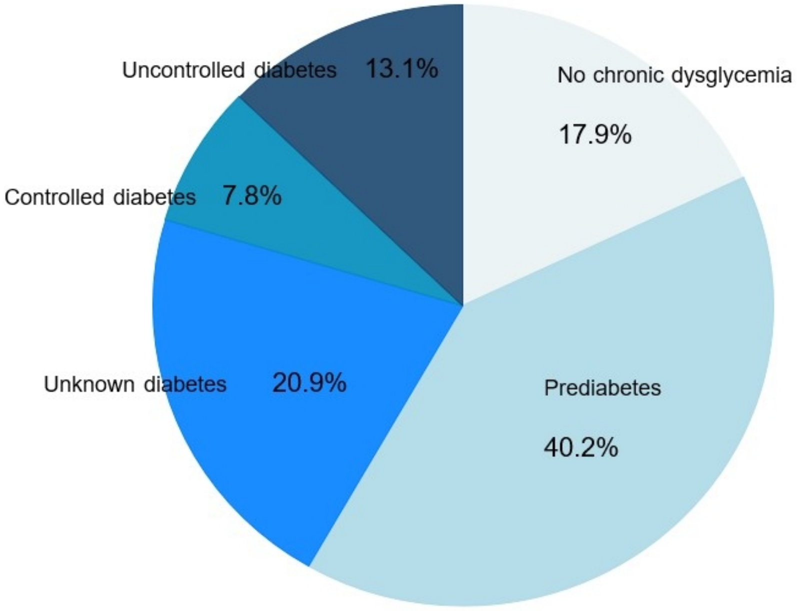

Figure 2. Prevalence of prediabetes, unknown diabetes and known diabetes among 206 consecutive ICU patients with Covid-19

144x102mm (144 x 144 DPI)

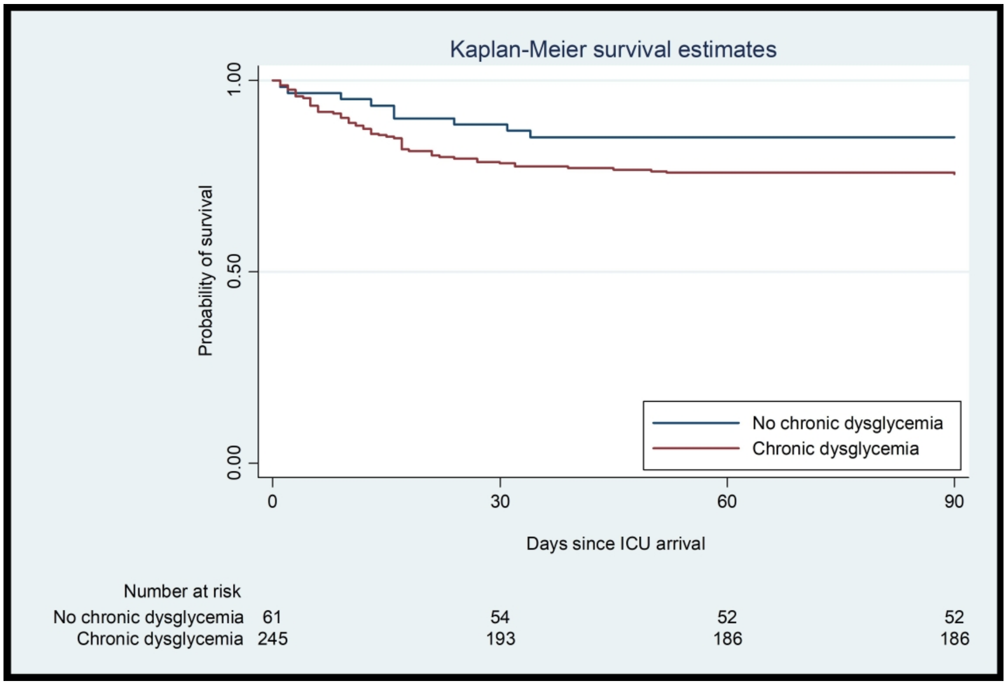

Figure 3. Probability of survival in no chronic dysglycemia patients and in patients with chronic dysglycemi

770x521mm (144 x 144 DPI)

Table S1: Post-hoc exploratory comparison between the subgroups for 90 days mortality and Renal replacement therapy

Subgroups	90 day mortality		Renal replacement therapy	
	N (%)	p	N (%)	p
No chronic dysglycemia vs Prediabetes	9/61 (14.7) vs 28/114 (24.5)	0.17	17/61 (27.8) vs 22/114 (19.2)	0.25
No chronic dysglycemia vs Unknown diabetes	9/61 (14.7) vs 12/60 (20.0)	0.48	17/61 (27.8) vs 11/60 (18.3)	0.28
No chronic dysglycemia vs Controlled diabetes	9/61 (14.7) vs 7/25 (28.0)	0.22	17/61 (27.8) vs 1/25 (4.0)	0.01
No chronic dysglycemia vs Uncontrolled diabetes	9/61 (14.7) vs 15/48 (31.2)	0.06	17/61(27.8) vs 8/48 (16.6)	0.25
Prediabetes vs Unknown diabetes	28/114 (24.5) vs 12/60 (20.0)	0.57	22/114 (19.2) vs 11/60 (18.3)	1
Prediabetes vs Controlled diabetes	28/114 (24.5) vs 7/25 (28.0)	0.79	22/114 (19.2) vs 1/ 25 (4.0)	0.07
Prediabetes vs Uncontrolled diabetes	28/114 (24.5) vs 15/48 (31.2)	0.43	22/114 (19.2) vs 8/48 (16.6)	0.8
Unknown diabetes vs Controlled diabetes	12/60 (20.0) vs 7/25 (28.0)	0.41	11/60 (18.3) vs 1/25 (4.0)	0.1
Unknown diabetes vs Uncontrolled diabetes	12/60 (20.0) vs 15/48 (31.2)	0.18	11/60 (18.3) vs 8/48 (16.6)	1
Controlled vs Uncontrolled diabetes	7/25 (28.0) vs 15/48 (31.2)	0.77	1/25 (4.0) vs 8/48 (16.6)	0.01
No chronic dysglycemia and prediabetes vs unknown and known diabetes	37/175 (21.1) vs 34/133 (25.5)	0.41	39/175 (22.2) vs 20/133 (15.0)	0.14

P values calculated with Fischer's exact test

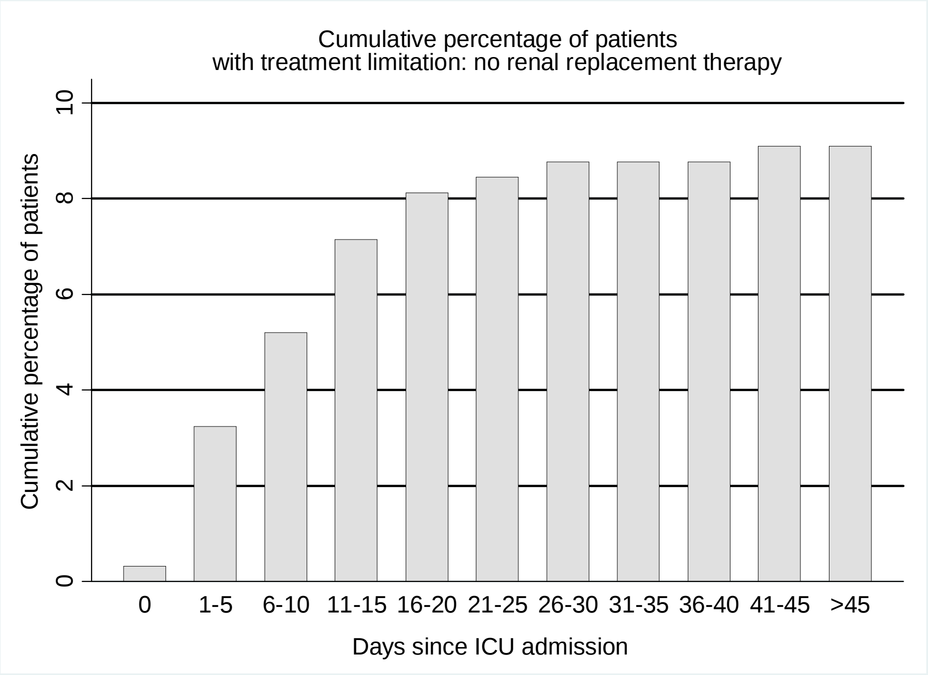

Figure S1. Cumulative percentage of patients with a treatment limitation: no renal replacement therapy. Day 0 (zero) denotes the day of admission to the intensive care unit.

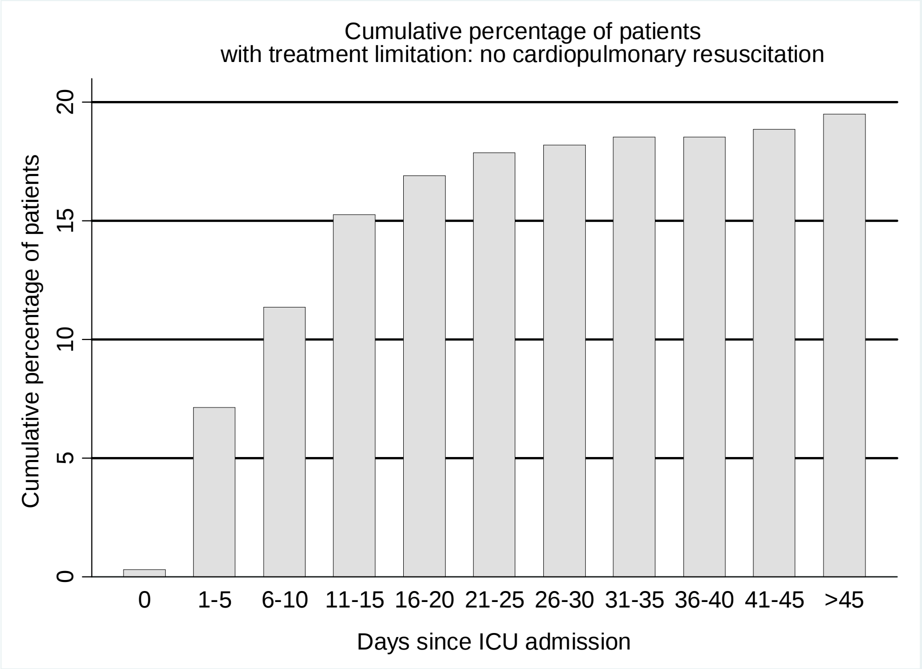

Figure S2. Cumulative percentage of patients with a treatment limitation: no cardiopulmonary resuscitation. Day 0 (zero) denotes the day of admission to the intensive care unit.

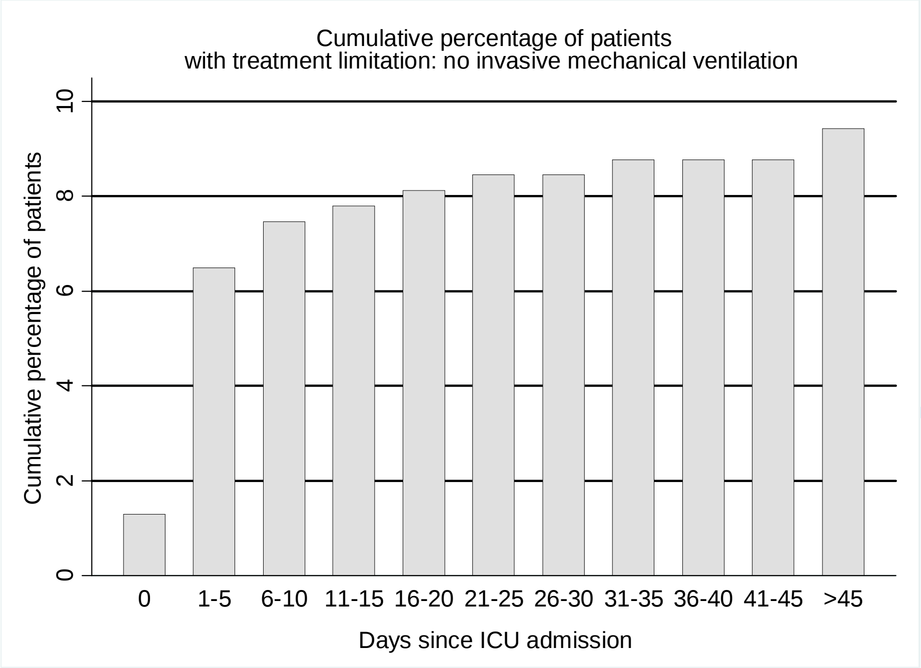

Figure S3. Cumulative percentage of patients with a treatment limitation: no invasive mechanical ventilation. Day 0 (zero) denotes the day of admission to the intensive care unit.

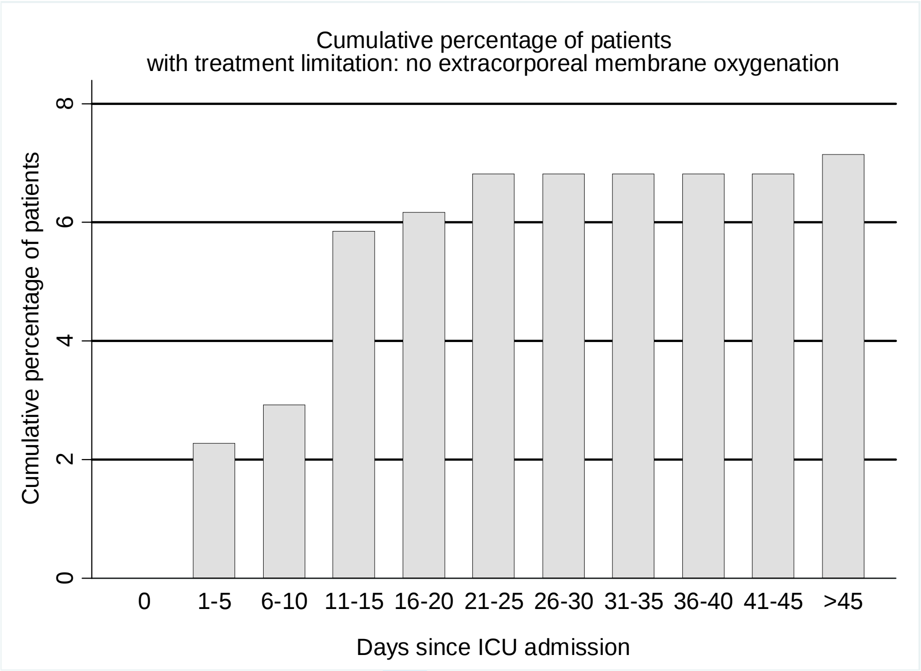

Figure S4. Cumulative percentage of patients with a treatment limitation: no extracorporeal membrane oxygenation. Day 0 (zero) denotes the day of admission to the intensive care unit.

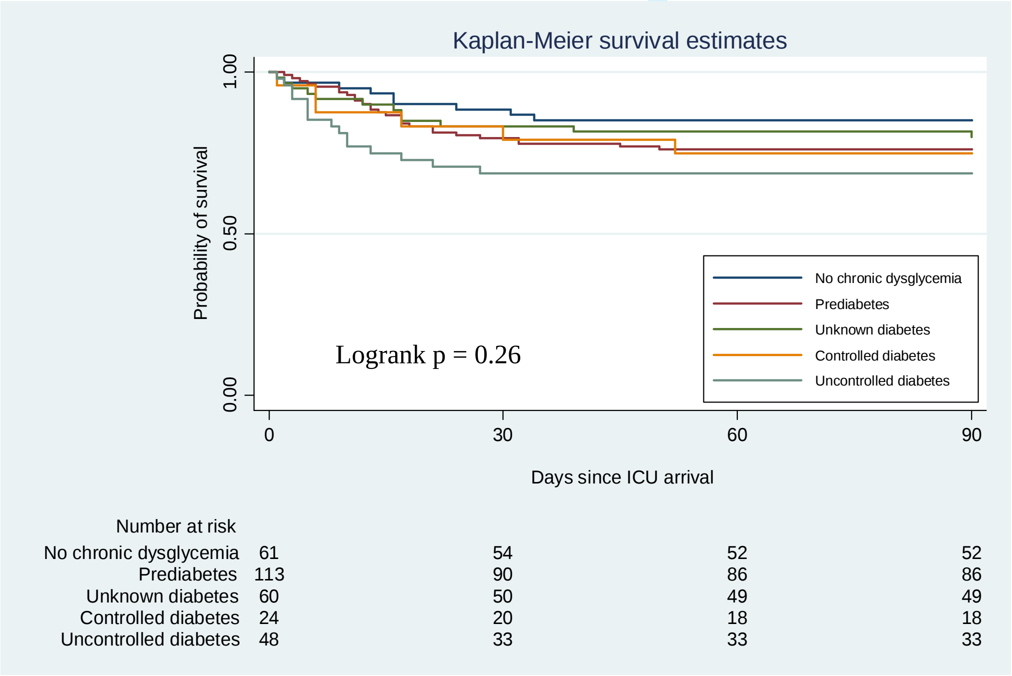

Figure S5. Probability of survival in the study groups

STROBE Statement—Checklist of items that should be included in reports of ***cohort studies***

Page references for document: **Chronic dysglycemia in critically ill patients with Covid-19 – a retrospective cohort study**

	Item No	Recommendation	Page No
**Title and abstract**	1	(*a*) Indicate the study's design with a commonly used term in the title or the abstract	1
		(*b*) Provide in the abstract an informative and balanced summary of what was done and what was found	2-3
**Introduction**			
Background/rationale	2	Explain the scientific background and rationale for the investigation being reported	3-4
Objectives	3	State specific objectives, including any prespecified hypotheses	4
**Methods4**			
Study design	4	Present key elements of study design early in the paper	4, 5
Setting	5	Describe the setting, locations, and relevant dates, including periods of recruitment, exposure, follow-up, and data collection	5-6
Participants	6	(*a*) Give the eligibility criteria, and the sources and methods of selection of participants. Describe methods of follow-up	5-7
		(*b*) For matched studies, give matching criteria and number of exposed and unexposed	NA
Variables	7	Clearly define all outcomes, exposures, predictors, potential confounders, and effect modifiers. Give diagnostic criteria, if applicable	7-8
Data sources/ measurement	8*	For each variable of interest, give sources of data and details of methods of assessment (measurement). Describe comparability of assessment methods if there is more than one group	5-7
Bias	9	Describe any efforts to address potential sources of bias	5
Study size	10	Explain how the study size was arrived at	5-6, Figure 1
Quantitative variables	11	Explain how quantitative variables were handled in the analyses. If applicable, describe which groupings were chosen and why	6-8
Statistical methods	12	(*a*) Describe all statistical methods, including those used to control for confounding	7
		(*b*) Describe any methods used to examine subgroups and interactions	7
		(*c*) Explain how missing data were addressed	8
		(*d*) If applicable, explain how loss to follow-up was addressed	NA
		(*e*) Describe any sensitivity analyses	NA
**Results**			
Participants	13*	(a) Report numbers of individuals at each stage of study—eg numbers potentially eligible, examined for eligibility, confirmed eligible, included in the study, completing follow-up, and analysed	8-9, Figure 1
		(b) Give reasons for non-participation at each stage	Figure 1
		(c) Consider use of a flow diagram	Figure 1

For peer review only - http://bmjopen.bmj.com/site/about/guidelines.xhtml

Descriptive data	14*	(a) Give characteristics of study participants (eg demographic, clinical, social) and information on exposures and potential confounders	8-9 Table 1
		(b) Indicate number of participants with missing data for each variable of interest	Table 1
		(c) Summarise follow-up time (eg, average and total amount)	NA
Outcome data	15*	Report numbers of outcome events or summary measures over time	10-11 Table 2-3

For peer review only

Main results	16	(*a*) Give unadjusted estimates and, if applicable, confounder-adjusted estimates and their precision (eg, 95% confidence interval). Make clear which confounders were adjusted for and why they were included<br>(*b*) Report category boundaries when continuous variables were categorized<br>(*c*) If relevant, consider translating estimates of relative risk into absolute risk for a meaningful time period	10-11 Table 3
Other analyses	17	Report other analyses done—eg analyses of subgroups and interactions, and sensitivity analyses	11 Figure 2,3,S1-4, S5
**Discussion**			
Key results	18	Summarise key results with reference to study objectives	11
Limitations	19	Discuss limitations of the study, taking into account sources of potential bias or imprecision. Discuss both direction and magnitude of any potential bias	14
Interpretation	20	Give a cautious overall interpretation of results considering objectives, limitations, multiplicity of analyses, results from similar studies, and other relevant evidence	15
Generalisability	21	Discuss the generalisability (external validity) of the study results	13-14
**Other information**			
Funding	22	Give the source of funding and the role of the funders for the present study and, if applicable, for the original study on which the present article is based	15

*Give information separately for exposed and unexposed groups.

Note: An Explanation and Elaboration article discusses each checklist item and gives methodological background and published examples of transparent reporting. The STROBE checklist is best used in conjunction with this article (freely available on the Web sites of PLoS Medicine at http://www.plosmedicine.org/, Annals of Internal Medicine at http://www.annals.org/, and Epidemiology at http://www.epidem.com/). Information on the STROBE Initiative is available at http://www.strobe-statement.org.

For peer review only

For peer review only - http://bmjopen.bmj.com/site/about/guidelines.xhtml

BMJ Open

Prevalence and impact of chronic dysglycemia among patients with Covid-19 in Swedish intensive care units: a multicenter, retrospective cohort study

Journal:	*BMJ Open*
Manuscript ID	bmjopen-2022-071330.R2
Article Type:	Original research
Date Submitted by the Author:	19-Aug-2023
Complete List of Authors:	Balintescu, Anca; Karolinska Institute, Department of Clinical Science and Education, Södersjukhuset<br>Rysz, Susanne; Karolinska Institute, Department of Perioperative Medicine and Intensive Care<br>Hertz, Carl; Stockholm South General Hospital Anaesthesia Department ICU<br>Grip, Jonathan; Karolinska Institute, Department of Perioperative Medicine and Intensive Care<br>Cronhjort, Maria; Karolinska Institute, Department of Clinical Science and Education Södersjukhuset<br>Oldner, Anders; Karolinska Institute, Department of Perioperative Medicine and Intensive Care<br>Svensen, Christer; Karolinska Institute, Department of Clinical Science and Education Södersjukhuset<br>Mårtensson, Johan; Karolinska Institute, Department of Perioperative Medicine and Intensive Care
**Primary Subject Heading**:	Intensive care
Secondary Subject Heading:	Diabetes and endocrinology
Keywords:	Adult intensive & critical care < INTENSIVE & CRITICAL CARE, COVID-19, DIABETES & ENDOCRINOLOGY, General diabetes < DIABETES & ENDOCRINOLOGY

SCHOLARONE™ Manuscripts

I, the Submitting Author has the right to grant and does grant on behalf of all authors of the Work (as defined in the below author licence), an exclusive licence and/or a non-exclusive licence for contributions from authors who are: i) UK Crown employees; ii) where BMJ has agreed a CC-BY licence shall apply, and/or iii) in accordance with the terms applicable for US Federal Government officers or employees acting as part of their official duties; on a worldwide, perpetual, irrevocable, royalty-free basis to BMJ Publishing Group Ltd ("BMJ") its licensees and where the relevant Journal is co-owned by BMJ to the co-owners of the Journal, to publish the Work in this journal and any other BMJ products and to exploit all rights, as set out in our licence.

The Submitting Author accepts and understands that any supply made under these terms is made by BMJ to the Submitting Author unless you are acting as an employee on behalf of your employer or a postgraduate student of an affiliated institution which is paying any applicable article publishing charge ("APC") for Open Access articles. Where the Submitting Author wishes to make the Work available on an Open Access basis (and intends to pay the relevant APC), the terms of reuse of such Open Access shall be governed by a Creative Commons licence – details of these licences and which Creative Commons licence will apply to this Work are set out in our licence referred to above.

Other than as permitted in any relevant BMJ Author's Self Archiving Policies, I confirm this Work has not been accepted for publication elsewhere, is not being considered for publication elsewhere and does not duplicate material already published. I confirm all authors consent to publication of this Work and authorise the granting of this licence.

For peer review only

Prevalence and impact of chronic dysglycemia among patients with Covid-19 in Swedish intensive care units: a multicenter, retrospective cohort study

Anca Balintescu, MD, PhD[1, 2]; Susanne Rysz, MD[3, 4]; Carl Hertz, MD[2]; Jonathan Grip, MD, PhD[3, 5]; Maria Cronhjort, MD, PhD[1, 2]; Anders Oldner MD, PhD[3, 6]; Christer Svensen MD, PhD[1, 2]; Johan Mårtensson, MD, PhD[3, 6]

[1] Department of Clinical Science and Education Karolinska Institute, Unit of Anesthesiology and Intensive Care, South General Hospital, Sjukhusbacken 10, 11883 Stockholm, Sweden

[2] Department of Anesthesia and Intensive Care, South General Hospital, Sjukhusbacken 10, 11883 Stockholm, Sweden

[3] Department of Perioperative Medicine and Intensive Care, Karolinska University Hospital, Norrbacka S2:05, SE-17176 Stockholm, Sweden

[4] Department of Medicine, Solna, Karolinska Institute, Stockholm, Sweden

[5] Department of Clinical Science, Intervention and Technology, Karolinska Institute, Stockholm, Sweden

[6] Department of Physiology and Pharmacology, Section of Anaesthesia and Intensive Care, Karolinska Institutet, Norrbacka S2:05, SE-17176 Stockholm, Sweden

Corresponding Author:

Anca Balintescu, ANOPIVA, South General Hospital, Sjukhusbacken 10, 11883 Stockholm, Sweden. Phone: +46 (0)722 7023 83. Fax: +46 (0)8-616 22 08. Email: anca.balintescu@ki.se

For peer review only - http://bmjopen.bmj.com/site/about/guidelines.xhtml

Keywords: HbA1c, ICU, Covid-19, prevalence, glycemic control, diabetes

Abstract

Objective: Using glycated hemoglobin A1c (HbA1c) screening, we aimed to determine the prevalence of chronic dysglycemia among patients with Covid-19 admitted to the intensive care unit (ICU). Additionally, we aimed to explore the association between chronic dysglycemia and clinical outcomes related to ICU stay.

Design: Multicenter retrospective observational study

Setting: ICUs in three hospitals in Stockholm, Sweden

Participants: Covid-19 patients admitted to the ICU between 5 March and 13 August 2020 with available HbA1c at admission. Chronic dysglycemia was determined based on previous diabetes history and HbA1c.

Primary and secondary outcomes: Primary outcome was the actual prevalence of chronic dysglycemia (prediabetes, unknown diabetes, or known diabetes) among Covid-19 patients. Secondary outcome was the association of chronic dysglycemia with 90-day mortality, ICU length of stay, duration of invasive mechanical ventilation (IMV), and renal replacement therapy (RRT), accounting for treatment selection bias.

Results: A total of 308 patients with available admission HbA1c were included. Chronic dysglycemia prevalence assessment was restricted to 206 patients admitted ICUs in which HbA1c was measured on all admitted patients. Chronic dysglycemia was present in 82.0% (95% CI 76.1%-87.0%) of patients, with prediabetes present in 40.2% (95% CI 33.5%-47.3%), unknown diabetes in 20.9% (95% CI 15.5%-27-1%), well-controlled diabetes in 7.8% (95% CI 4.5%-12.3%), and uncontrolled diabetes in 13.1% (95% CI 8.8%-18.5%). All patients with available HbA1c were included for the analysis of the relationship between chronic dysglycemia and secondary outcomes.

For peer review only - http://bmjopen.bmj.com/site/about/guidelines.xhtml

We found no independent association between chronic dysglycemia and 90-day mortality, ICU length of stay, or duration of IMV. After excluding patients with specific treatment limitations, no association between chronic dysglycemia and RRT use was observed.

Conclusions: In our cohort of critically ill Covid-19 patients, the prevalence of chronic dysglycemia was 82%. We found no robust associations between chronic dysglycemia and clinical outcomes when accounting for treatment limitations.

Strengths and limitations of this study

- Presents prevalence of chronic dysglycemia in an ICU population with Covid-19 based on additional quantification of admission HbA1c
- Actual prevalence of chronic dysglycemia calculation in all ICU admitted patients, reducing the risk of ascertainment bias
- Treatment limitations were considered in the analysis of clinical outcomes, thereby reducing the risk of treatment selection bias.
- We lack data on glycemic control during ICU stay, that might have influenced clinical outcomes

Background

Diabetes has been identified as a frequent comorbidity in patients with Covid-19, with a prevalence ranging from 7.4% to 34.3% among those requiring hospitalization [1-3]. A meta-analysis published in April 2020 found diabetes to be the second most frequent comorbidity in patients with Covid-19 admitted to the intensive care unit

For peer review only - http://bmjopen.bmj.com/site/about/guidelines.xhtml

(ICU) [4]. Furthermore, Covid-19 patients with diabetes appear to have a significantly higher risk of ICU admission and worse prognosis than Covid-19 patients without diabetes [4-6]. Particularly, a glycated hemoglobin A1c (HbA1c) level above 7% (53 mmol/mol) was identified as risk factor for ICU admission [7].

Recent data also indicates that diabetes is associated with worse prognosis among ICU patients with Covid-19 [8]. However, these studies did not include HbA1c measurements to identify patients with prediabetes or previously undiagnosed diabetes. This is an important limitation since both prediabetes and diabetes is considerably under-diagnosed both in the community [9] and in the ICU [10]. Additionally, a history of diabetes diagnosis and HbA1c at ICU admission, measured in consecutively admitted patients, is important in determining the true prevalence of chronic dysglycemia in the critically ill Covid-19 population. Finally, information about limitations of life-sustaining treatment were not considered in previous outcome analyses. This is unfortunate since the presence of such limitations may introduce treatment selection bias.

We therefore conducted a multicenter observational study using quantification of HbA1c and information about diabetes history to determine the actual prevalence of chronic dysglycemia (prediabetes, unknown diabetes, or known diabetes) among Covid-19 patients admitted to ICU. In addition, we aimed to explore the relationship of chronic dysglycemia with 90-day mortality, ICU length of stay, duration of invasive mechanical ventilation (IMV) and severe acute kidney injury requiring renal replacement therapy (RRT) accounting for treatment selection bias. We hypothesised that prevalence of chronic dysglycemia in Covid-19 patients admitted to the ICU exceeds the prevalence of chronic dysglycemia in the non Covid-19 critically ill

For peer review only - http://bmjopen.bmj.com/site/about/guidelines.xhtml

population. Moreover, we hypothesised that such chronic dysglycemia would be associated with worse clinical outcomes during ICU stay in patients with Covid-19.

Material and Methods

The study was approved by the Swedish Ethical Review Authority (approval number 2020-01302, amendment 2020-02890) with a waiver of informed consent. The study was performed in accordance with the Helsinki Declaration and reported in conformity with the STROBE statement [11]

Patient and Public Involvement statement: The study is based on data that was collected during the ongoing Covid-19 pandemic in a quality register. No intervention was applied to the individual patient. The public and patients were not involved in the design of the study. Results are to be disseminated to the public and scientific community through publication in peer-reviewed journal with open access.

Study design

We conducted a multicenter, retrospective observational study of adult (≥18 years) patients with a positive polymerase chain reaction (PCR) for severe acute respiratory syndrome coronavirus 2 (SARS-CoV-2) admitted to ten ICUs in three hospitals in Stockholm, Sweden between March 5th and August 13th, 2020 (first wave). We excluded patients without HbA1c obtained on admission to the ICU, patients in the third trimester of pregnancy and patients with a primary admission diagnosis other than Covid-19. In patients with multiple ICU admissions, only the first admission was considered. All included patients were assessed in the outcome analyses.

Assessment of chronic dysglycemia prevalence was restricted to a nested cohort of

For peer review only - http://bmjopen.bmj.com/site/about/guidelines.xhtml

patients from ICUs in which HbA1c measurement was included in the routine laboratory panel performed on all consecutive admissions. In the prevalence analysis, we therefore excluded patients with available HbA1c who were admitted to ICUs in which HbA1c was measured only at the discretion of the treating clinicians.

Data collection

HbA1c was measured in whole blood at ICU admission using the VARIANT II TURBO Hemoglobin Testing System analyzer (Bio-Rad Laboratories GmBH) and was reported in mmol/mol (IFCC calibrated) and in %. HbA1c was measured as part of routine care in three ICUs and at the discretion of the treating clinician in seven ICUs. We collected information on demographics, comorbidity, chronic medication, HbA1c value, mortality and decision regarding limitation of life-sustaining care from the patients' medical records (Take Care [CompuGroup Medical, Koblenz, Germany]). International Classification of Disease (ICD) 10 codes were used to identify comorbidity and previous history of diabetes. Additionally, data regarding known diabetes diagnosis was extracted manually from the patients' medical records. Data on body mass index (BMI), Simplified Acute Physiology Score (SAPS) 3, ICU length of stay, duration of IMV and RRT were collected from the ICU electronic patient data management system Clinisoft (GE, Barringgton, IL).

Prediabetes and Diabetes definitions

Prediabetes and diabetes were diagnosed based on two complementary methods; level of HbA1c at admission and previous medical history of diabetes, and categorized into five groups:

(1) no diabetes (HbA1c <42 mmol/mol [6.0%] and no history of diabetes)

For peer review only - http://bmjopen.bmj.com/site/about/guidelines.xhtml

(2) prediabetes (HbA1c 42-47 mmol/mol [6.0-6.4%] and no history of diabetes)

(3) unknown diabetes (HbA1c ≥48 mmol/mol [6.5 %] and no history of diabetes)

(4) controlled diabetes (HbA1c <52 mmol/mol [6.9 %] and previous history of diabetes)

(5) uncontrolled diabetes (HbA1c ≥52 mmol/mol [6.9 %] and previous history of diabetes).

In Sweden, the diagnosis of prediabetes and diabetes is based on the World Health Organisation's (WHO) HbA1c cut off values [12], not the American Diabetes Association's (ADA). Therefore, we used the WHO criteria to classify the study groups in our research.

Individuals in group (2), (3), (4) and (5) were considered to have chronic dysglycemia compared to those in group (1) labeled "no chronic dysglycemia".

Outcomes

The primary outcome was the prevalence of chronic dysglycemia. Secondary outcomes included 90-day mortality, ICU length of stay, duration of IMV, and RRT use.

Statistical Analysis

We analyzed data using STATA version 12.1 (Stata Corp., College Station, Texas). Categorical data is presented as numbers and percentages and compared using the Fisher's exact test. Continuous data is summarized as median with interquartile range (IQR) and compared using the Mann-Whitney U test. The prevalence of chronic dysglycemia (primary outcome) was presented as percentages with 95% confidence intervals (CI). We displayed time to death within 90 days using Kaplan-

For peer review only - http://bmjopen.bmj.com/site/about/guidelines.xhtml

Meier curves. Survival curves were compared using a log-rank test. We used multivariable Cox regression analysis to assess the association between chronic dysglycemia and 90-day mortality. We used multivariable linear regression analysis to assess the association with ICU length of stay and duration of IMV. Both these outcomes were found to be well approximated by log-normal distributions and were therefore log-transformed before analysis with results presented as geometric means (95% CI). We used multivariable logistic regression analysis to assess the association with RRT use, before and after excluding patients with RRT as a treatment limitation. All regression analyses were conducted using the following models: adjusted for SAPS 3, age and sex, and adjusted for SAPS 3, age, sex, hypertension, any malignancy, any treatment limitation on admission and chronic corticosteroid use. A post-hoc exploratory comparison between subgroups was done for 90 day mortality and RRT use. A two-sided P-value <0.05 was considered statistically significant.

Results

Patients

A total of 584 patients with positive SARS-CoV-2 test were admitted to the study ICUs during the study period. We excluded 225 patients without available HbA1c, six pregnant patients, 16 readmissions and 29 patients without symptoms associated with Covid-19. Therefore, we included 308 patients with available HbA1c for outcome analysis. Among those 308 patients, 206 consequently admitted patients in which HbA1c was included in the admission routine laboratory panel were used for prevalence calculation (Figure 1). Baseline characteristics and treatment limitations of the entire study population are detailed in Table 1.

For peer review only - http://bmjopen.bmj.com/site/about/guidelines.xhtml

Table 1. Baseline characteristics and treatment limitations

		Chronic dysglycemia				
**Characteristic**	**No chronic dysglycemia**	**Prediabetes**	**Unknown diabetes**	**Controlled diabetes**	**Uncontrolled diabetes**	**P[e]**
No. (%)	61 (19.8)	114 (37.0)	60 (19.4)	25 (8.11)	48 (15.5)	
Age, years	57 (51,63)	61 (53, 68)	60 (52, 68)	63 (57, 71)	62 (55,69)	0.03
Male sex	48 (78.6)	92 (80.7)	47 (78.3)	21 (84.0)	36 (75.0)	1.00
Body mass index[a], kg/m^2	27 (25, 32)	27 (25, 30)	28 (25, 31)	29 (26, 32)	30 (26, 33)	0.97
HbA1c, mmol/mol	39 (36, 40)	44 (43, 46)	51 (49, 57)	47 (44, 49)	70 (61, 81)	<0.001
Diabetes treatment						
Diet only				6 (24.0)	2 (4.1)	
OAD only				17 (68)	19 (39.5)	
Insulin only				1 (4.0)	12 (25.0)	
OAD+Insulin				1 (4.0)	15 (31.2)	
Comorbidity						
Hypertension	18 (29.5)	40 (35.0)	23 (38.3)	16 (64.0)	34 (70.8)	0.02
Heart failure	6 (9.8)	5 (4.3)	6 (10.0)	0 (0.0)	3 (6.2)	0.24
Previous myocardial infarction	2 (3.2)	4 (3.5)	6 (10.0)	0 (0.0)	7 (14.5)	0.38
Chronic kidney disease	4 (6.5)	13 (11.4)	7 (11.6)	6 (24.0)	11 (22.9)	0.09
Liver disease	2 (3.2)	4 (3.5)	1 (1.6)	1 (4.0)	1 (2.0)	1.00
Any malignancy	0 (0.0)	8 (7.0)	2 (3.3)	2 (8.0)	4 (8.3)	0.04
Astma/COPD	13 (21.3)	20 (17.5)	14 (23.3)	5 (20.0)	9 (18.7)	0.72
SAPS 3[b]	53 (48, 60)	55 (49, 60)	57 (52, 62)	59 (52, 63)	56 (52, 69)	0.18
Chronic drug use						
Corticosteroids[c]	5 (8.20)	16 (14.04)	8 (13.3)	4 (16.0)	6 (12.5)	0.24
Immunosuppressive therapy[d]	1 (1.6)	8 (7.0)	3 (5.0)	1 (4.0)	1(2.0)	0.31
Treatment limitations[f]						
Any limitation	14 (22.9)	19 (16.6)	13 (21.6)	8 (32.0)	13 (27.0)	0.86
No RRT	5 (8.2)	7 (6.1)	5 (8.3)	5 (20.0)	6 (12.5)	1.00
No IMV	6 (9.8)	10 (8.7)	4 (6.6)	4 (16.0)	6 (12.5)	1,00
No CPR	9 (14.7)	19 (16.6)	12 (20.0)	8 (32.0)	12 (25.0)	0.36
No ECMO	7 (11.4)	3 (2.6)	5 (8.3)	3 (12.0)	3 (6.2)	0.15
Palliative care[g]	1 (1.6)	18 (16.5)	7 (12.2)	4 (16.6)	5 (10.8)	0.006

Data are n (%) or median (interquartile range)
HbA1c, glycated hemoglobin A1c; OAD, oral hypoglycemic agent; COPD, Chronic obstructive pulmonary disease; SAPS, Simplified Acute Physiology Score, 2 missing values (306 patients with data); RRT, renal replacement therapy; IMV, invasive mechanical ventilation; CPR, cardiopulmonary resuscitation
[a]Missing data in 15 patients (293 patients with data)
[b]Missing data in 2 patients (306 patients with data)
[c]Systemic or inhalatory corticosteroids
[d]Immunosuppressive therapy was defined as: treatment with Metotrexate, Azatioprin, Ciklosporin, Tracolimus, Infliximab
[e]P values for the comparison between no chronic dysglycemia and chronic dysglycemia, Mann-Whitney U test was used for comparison of continuous data and Fischer's exact test for comparison of categorical data
[f] Decision taken any time during ICU stay
[g]Decision to go over to palliative care taken during ICU stay

For peer review only - http://bmjopen.bmj.com/site/about/guidelines.xhtml

Patients with chronic dysglycemia were older, were more likely to have hypertension, malignancy and/or chronic kidney disease, and had higher SAPS 3 than patients without chronic dysglycemia. Overall, 14 (22.9%) patients in the no chronic dysglycemia group and 53 (21.5%) patients in the chronic dysglycemia group received one or more limitations of life-supporting therapies during their ICU stay. "No Cardiopulmonary resuscitation (CPR)" was the most common treatment limitation. We observed the highest proportion of limitations among patients with known (controlled or uncontrolled) diabetes. Decision to switch to palliative care was made in 1 (1.6%) patient in the no chronic dysglycemia group and 34 (13.8%) patients in the chronic dysglycemia group (P=0.006). Cumulative percentage of treatment limitations relative ICU admission is displayed in Figures S1-S4.

Primary outcome

In the nested cohort of 206 consecutive patients with available HbA1c, 169 (82.0%; 95% CI 76.1%-87.0%) were diagnosed with chronic dysglycemia. Prediabetes was present in 83 (40.2%, 95% CI 33.5%-47.3%), unknown diabetes in 43 (20.9%, 95% CI 15.5%-27-1%), well-controlled diabetes in 16 (7.8%, 95% CI 4.5%-12.3%), and uncontrolled diabetes in 27 (13.1%, 95% CI 8.8%-18.5%) patients (Figure 2).

Secondary outcomes

Nine (14.7%) patients in the no chronic dysglycemia group and 62 (25.1%) patients in the chronic dysglycemia group died within 90 days (P=0.09) (Table 2, Figure 3 and Figure S5). ICU length of stay and duration of IMV were similar in the two groups. IMV was delivered to 42 (68.8%) patients without chronic dysglycemia and to 187 (75.7%) patients with chronic dysglycemia (P=0.32). RRT was delivered to 17

For peer review only - http://bmjopen.bmj.com/site/about/guidelines.xhtml

(27.9%) no chronic dysglycemia patients and 42 (17.0%) chronic dysglycemia patients (P=0.06) (Table 2 and Table 3).

Table 2. Secondary outcomes

		Chronic dysglycemia				
**Outcomes**	**No chronic dysglycemia (n = 61)**	**Prediabetes (n = 114)**	**Unknown diabetes (n =60)**	**Controlled diabetes (n = 25)**	**Uncontrolled diabetes (n = 48)**	**P[a]**
90-day mortality, n (%)	9 (14.7)	28 (24.5)	12 (20.0)	7 (28.0)	15 (31.2)	0.09
ICU length of stay, days	9 (4, 25)	14 (6, 24)	13 (6, 28)	8 (5, 21)	11 (7, 22)	0.69
Invasive mechanical ventilation, days	16 (8, 29)	14 (10, 23)	15 (10, 27)	15 (6, 21)	14 (9, 22)	0.60
Renal replacement therapy, n (%)	17 (27.9)	22 (19.3)	11 (18.3)	1 (4.0)	8 (16.6)	0.06

Data are n (%) or median (interquartile range)
ICU lengths of stay (4 missing values because of transfer to other hospital)
[a] P values for the comparison between no chronic dysglycemia and chronic dysglycemia, Mann-Whitney U test was used for comparison of continuous data and Fischer's exact test for comparison of categorical data.

Table 3. Multivariable regression analyses showing the association of chronic dysglycemia (versus no chronic dysglycemia) with secondary outcomes

**Outcome measure**	**No Chronic Dysglycemia**	**Chronic Dysglycemia**	**Adjusted Risk Estimate (95% CI)[a]**	**P[a]**	**Adjusted Risk Estimate (95% CI)[b]**	**P[b]**	**Statistical test**
**90-day mortality n (%)**	9/61 (14.7)	62/247 (25.1)	1.61 (0.79 to 3.26)	0.18	1.54 (0.74 to 3.19)	0.24	Cox regression
**ICU length of stay, days**							
All patients	9 (4, 25)	13 (6, 23)	1.06 (0.78 to 1.43)	0.70	1.05 (0.77 to 1.44)	0.71	Linear regression
ICU survivors[c]	9 (5, 27)	14 (7, 24)	1.04 (0.76 to 1.43)	0.75	1.05 (0.76 to 1.44)	0.75	Linear regression
**Invasive mechanical ventilation duration, days**							
All patients[d]	16 (8, 29)	14 (10, 23)	0.92 (0.68 to 1.23)	0.58	0.93 (0.69 to 1.24)	0.63	Linear regression
ICU survivors[e]	16 (8, 30)	15 (10, 23)	0.93 (0.70 to 1.23)	0.61	0.92 (0.70 to 1.22)	0.59	Linear regression
**Renal replacement therapy, n (%)**							

For peer review only - http://bmjopen.bmj.com/site/about/guidelines.xhtml

All patients	17/61 (27.9)	42/247 (17.0)	0.52 (0.26 to 1.02)	0.06	0.49 (0.24 to 0.99)	0.04	Logistic regression
Patients without treatment limitation as no RRT	17/57 (29.8)	42/224 (18.8)	0.52 (0.26 to 1.04)	0.10	0.52 (0.25 to 1.07)	0.08	Logistic regression

[a]Multivariable models were adjusted for Simplified Acute Physiology Score (SAPS) 3, age and sex.
[b] Multivariable models were adjusted for Simplified Acute Physiology Score (SAPS) 3, age, sex, hypertension, any malignancy, any treatment limitation on admission and chronic corticosteroid use
[c]ICU length of stay in ICU survivors, 260 observations
[d]Invasive mechanical ventilation duration, 227 observations
[e]Invasive mechanical ventilation duration in ICU survivors, 189 observations

On multivariable regression analysis we observed a numerically higher mortality (adjusted HR 1.54, 95% CI 0.74-3.19, P=0.24) and significantly lower RRT use (adjusted OR 0.49, 95% CI 0.24-0.99, P=0.04) in patients with chronic dysglycemia (Table 3). No association with RRT was observed after exclusion of patients with "No RRT" as treatment limitation. In the post-hoc exploratory comparison between subgroups, RRT use was higher in the no diabetes group compared to the controlled diabetes group, as well as in the uncontrolled diabetes compared to controlled diabetes group (Table S1). Individuals with uncontrolled diabetes had the lowest probability of survival followed by individuals with controlled diabetes and prediabetes. The highest probability of survival was observed among patients with no chronic dysglycemia and prediabetes, respectively (Figure S5). However, we observed no statistically significant differences in mortality in the post-hoc comparison of subgroups (Table S1).

Discussions

Key findings

We performed a multicenter observational investigation to determine the prevalence of chronic dysglycemia and its impact on clinical outcomes among Covid-19 patients

For peer review only - http://bmjopen.bmj.com/site/about/guidelines.xhtml

admitted to ICU. Using available information about the patients' diabetic status in combination with routine HbA1c assessment, we found that 82% had chronic dysglycemia with two thirds having either prediabetes or undiagnosed diabetes. We observed numerically higher 90-day mortality in patients with chronic dysglycemia, with the highest mortality (31%) observed among those with uncontrolled diabetes. Conversely, the proportion of patients receiving RRT was lower among patients with chronic dysglycemia even when patients without "No RRT" as treatment limitation were considered separately. We found no association of chronic dysglycemia with ICU length of stay or duration of IMV.

Relationship with previous studies

A global meta-analysis of more than 16000 ICU patients with Covid-19 suggests a pooled prevalence of known diabetes between 23-31% [13], close to the observed prevalence in our study (21%). However, few studies have used additional HbA1c measurements to assess the actual prevalence of chronic dysglycemia, including prediabetes and undiagnosed diabetes. One such ICU study from Austria found a prevalence of chronic dysglycemia of 85%, which is in close agreement with our findings [14]. However, the Austrian study did not assess consecutive patients and may therefore be prone to selection bias.

Our findings indicate that chronic dysglycemia is more common in Covid-19 patients than in ICU patients with other admission diagnoses. In fact, in a pre-Covid-19 cohort of general ICU patients we found a corresponding dysglycemia prevalence of 33% [10]. The relationship between severe SARS-CoV-2 infection and dysglycemia has different potential explanations. SARS-CoV-2 enters cells in various organs, including the pancreas, via angiotensin-converting enzyme II (ACE2). As ACE2 is involved in

For peer review only - http://bmjopen.bmj.com/site/about/guidelines.xhtml

regulating pancreatic beta-cell function, a link between SARS-CoV-2 infection and beta-cell dysfunction and diabetes development has been suggested [15]. Interestingly, the prevalence of elevated HbA1c below the diabetes diagnostic threshold (prediabetes) was markedly higher in our Covid-19 cohort than in our previous pre-Covid-19 cohort (40% vs 9%). It is possible that the duration of Covid-19 symptoms before ICU admission (typically ten days in the literature [16]) was sufficient to trigger new onset hyperglycemia with mildly elevated HbA1c.

In addition to the above speculations about SARS-CoV-2 as a *cause* of dysglycemia, there is also evidence suggesting that patients with preexisting dysglycemia are prone to a more severe course of Covid-19. For example, some studies have shown that hospitalized SARS-CoV-2 positive with prediabetes, unknown diabetes, and known poorly controlled diabetes are at increased risk of SARS-CoV-2 associated respiratory failure requiring intensive care [17]. A higher burden of comorbidities, hyperglycemia *per se*, and chronic low-grade inflammation in diabetes may explain this observation [18].

Wang et al identifies fasting glucose as an independent predictor for 28-day mortality in hospitalized individuals with Covid-19 and previously unknown diabetes. However, HbA1c was not assessed and interference from stress hyperglycemia might have led to the different results compared to our study [19].

Others [20], identified an increased risk of death in individuals with diabetes and increasing levels of HbA1c above 48 mmol/mol and known diabetes in a large cohort of hospitalized patients, but not in critically ill individuals.

Whether chronic dysglycemia is associated with worse outcomes among Covid-19 patients admitted to ICU remains uncertain. Dennis et al [21] found increased mortality risk at 30 days (HR 1.23 [95% CI 1.14, 1.32]) compared to patients with no

For peer review only - http://bmjopen.bmj.com/site/about/guidelines.xhtml

diabetes in patients admitted to the high Dependency Unit or ICU, but did not take HbA1c into consideration. A multicenter study from France including 410 ICU patients with Covid-19, found no association between the severity of dysglycemia and tracheal intubation and/or death within 7 days of admission in patients with diabetes than in those without diabetes [22]. This is in accordance with the findings of our study. In contrast, others found higher mortality in the subgroup of mechanically ventilated patients with diabetes [14].

We previously demonstrated an independent association between chronic dysglycemia and need for RRT in critically ill non-Covid-19 patients [10]. This association was, however, not found in the present study. In fact, we observed a higher proportion of patients requiring RRT among our patients without chronic dysglycemia and an inverse association between chronic dysglycemia and RRT use. Only one individual in the controlled diabetes subgroup received RRT during ICU stay. We believe this surprising finding may be due to treatment limitations. In fact, after exclusion of patients with treatment limitation "not for RRT", we observed no statistically significant association between chronic dysglycemia and RRT use. Limitations in life-sustaining care were more common in the known diabetes groups (well controlled and uncontrolled diabetes) than in all other groups. We cannot exclude the possibility that patients with severe acute or chronic kidney injury did not reach the ICU because of treatment limitation decisions made at hospital arrival or on the medical ward. This might have influenced the number of patients with kidney injury reaching the ICU, affecting predominantly patients with chronic dysglycemia, as they are usually older and have multiple comorbidities.

Strengths and limitations

For peer review only - http://bmjopen.bmj.com/site/about/guidelines.xhtml

Our study has several strengths. It is the first to assess the prevalence of chronic dysglycemia in an ICU population with Covid-19 based on additional quantification of admission HbA1c. This approach reduced bias due to events that would have influenced HbA1c values obtained before ICU admission. We restricted the prevalence assessment to a cohort of patients who were admitted to ICUs where HbA1c was part of the routine laboratory panel, thereby reducing the risk of ascertainment bias. Additionally, by measuring HbA1c in all patients admitted to the ICU we identified 169 (82%) individuals with chronic dysglycemia and 86 (41.7%) with diabetes. If HbA1c would not have been measured routinely at ICU admission, we would only have identified 43 (20.9%) individuals with diabetes. Furthermore, we considered treatment limitations in our analysis of clinical outcomes, thereby reducing the risk of treatment selection bias. Finally, we included patients admitted to ten ICUs in three University hospitals, thus providing a degree of external validity for applying our findings to similar settings.

Our study has limitations. We lack data on conditions and treatment that might have influenced admission HbA1c, such as haemoglobinopathies and blood transfusion before ICU admission. Since interviews with patients or relatives were not performed, a degree of misclassification due to non-documented dysglycemia diagnoses cannot be ruled out. However, such interviews would have been logistically difficult during the ongoing pandemic. We used an HbA1c cutoff of 42-47 mmol/mol (6.0-6.4%) to classify prediabetes. If we instead had used the cutoff suggested by the ADA (39-47 mmol/mol [5.7-6.4%], our prevalence of chronic dysglycemia would have increased from 82.0% to 91.3%. This approach did not, however, alter the association with the secondary outcomes (data not shown). In addition, we lack information about glycemic control during intensive care, which might have modified clinical outcomes.

For peer review only - http://bmjopen.bmj.com/site/about/guidelines.xhtml

The observational nature of the study does not imply causation. Generalizability of our results is limited to populations with similar health care systems and similar legal frame-works for decisions on treatment limitations. Finally, the limited sample size may limit the conclusion regarding secondary outcomes that can be drawn from the data.

Conclusion

In our multicenter cohort of Covid-19 patients admitted to the ICU, HbA1c screening diagnosed chronic dysglycemia in four out of five patients with the majority having either prediabetes or previously undiagnosed diabetes. Chronic dysglycemia was not significantly associated with mortality, ICU length of stay, duration of invasive mechanical ventilation or renal replacement therapy use after considering treatment limitations. These findings indicate that chronic dysglycemia may be a risk factor for severe Covid-19. However, Covid-19 prognosis in the ICU does not appear to be modified by chronic dysglycemia.

Author Contributions:

AB, SR, MC, JG, AO, CS and JM contributed to the concept and design of the study.

AB, SR, CH and JM collected data.

AB, SR, CH, JG, MC, CS, AO and JM contributed to the analysis and interpretation of data.

AB and JM drafted the manuscript.

All authors critical reviewed and approved the final manuscript.

For peer review only - http://bmjopen.bmj.com/site/about/guidelines.xhtml

AB accepts full responsibility for the work and the conduct of the study, had access to the data, and controlled the decision to publish.

Funding AB and JM were supported by Region Stockholm (clinical research appointment and ALF project grants), grant number 580282.

A non-peer-reviewed version of this article has been a part of a publicly defended PhD thesis ISBN 978-91-8016-719-2 and was submitted to Karolinska Institute Open Archive, available at https://openarchive.ki.se/xmlui/handle/10616/48203 on 19 September 2022.

Competing interest: The authors have no competing interest relevant to this work.

Data availability: The data that support the findings of this study are available from the corresponding author upon reasonable request.

Ethics Approval Statement: The study was approved by the Swedish Ethical Review Authority (approval number 2020-01302).

References

[1] Yang J, Zheng Y, Gou X, Pu K, Chen Z, Guo Q, et al. Prevalence of comorbidities and its effects in patients infected with SARS-CoV-2: a systematic review and meta-analysis. International Journal of Infectious Diseases 2020;94:91-5. doi:10.1016/j.ijid.2020.03.017

[2] Liu Y, Lu R, Wang J, Cheng Q, Zhang R, Zhang S, et al. Diabetes, even newly defined by HbA1c testing, is associated with an increased risk of in-hospital death in adults with COVID-19. BMC Endocrine Disorders 2021;21(1):1-10. doi:10.1186/s12902-021-00717-6

[3] Guan W-j, Liang W-h, Zhao Y, Liang H-r, Chen Z-s, Li Y-m, et al. Comorbidity and its impact on 1590 patients with COVID-19 in China: a nationwide analysis. European Respiratory Journal 2020;55(5):2000547. doi:10.1183/13993003.00547-2020

[4] Roncon L, Zuin M, Rigatelli G, Zuliani G. Diabetic patients with COVID-19 infection are at higher risk of ICU admission and poor short-term outcome. J Clin Virol 2020;127:104354. doi:10.1016/j.jcv.2020.104354

For peer review only - http://bmjopen.bmj.com/site/about/guidelines.xhtml

[5] Zhou F, Yu T, Du R, Fan G, Liu Y, Liu Z, et al. Clinical course and risk factors for mortality of adult inpatients with COVID-19 in Wuhan, China: a retrospective cohort study. The Lancet 2020;395. doi:10.1016/S0140-6736(20)30566-3

[6] Guo W, Li M, Dong Y, Zhou H, Zhang Z, Tian C, et al. Diabetes is a risk factor for the progression and prognosis of COVID-19. Diabetes/metabolism research and reviews 2020:e3319.

[7] Lei M, Lin K, Pi Y, Huang X, Fan L, Huang J, et al. Clinical Features and Risk Factors of ICU Admission for COVID-19 Patients with Diabetes. Journal of Diabetes Research 2020;2020:5237840. doi:10.1155/2020/5237840

[8] Grasselli G, Greco M, Zanella A, Albano G, Antonelli M, Bellani G, et al. Risk Factors Associated With Mortality Among Patients With COVID-19 in Intensive Care Units in Lombardy, Italy. JAMA internal medicine 2020;180(10):1345-55. doi:10.1001/jamainternmed.2020.3539

[9] Beagley J, Guariguata L, Weil C, Motala AA. Global estimates of undiagnosed diabetes in adults. Diabetes Research and Clinical Practice 2014;103(2):150-60. doi:https://doi.org/10.1016/j.diabres.2013.11.001

[10] Balintescu A, Palmgren I, Lipcsey M, Oldner A, Larsson A, Cronhjort M, et al. Prevalence and impact of chronic dysglycemia in intensive care unit patients-A retrospective cohort study. Acta Anaesthesiol Scand 2021;65(1):82-91. doi:10.1111/aas.13695

[11] Erik von Elm DGA, Matthias Egger, Stuart J. Pocock, Peter C. Gøtzsche, and Jan P. Vandenbroucke. The Strengthening the Reporting of Observational Studies in Epidemiology (STROBE) Statement: Guidelines for Reporting Observational Studies. Annals of Internal Medicine 2007;147(8):573-7. doi:10.7326/0003-4819-147-8-200710160-00010 %m 17938396

[12] Organisation WH. World Health Organisation. Use of Glycated Haemoglobin (HbA1c) in the Diagnosis of Diabetes Mellitus, https://www.who.int/publications/i/item/use-of-glycated-haemoglobin-(-hba1c)-in-diagnosis-of-diabetes-mellitus; [accessed 07 Fenruary 2022].

[13] Tan E, Song J, Deane AM, Plummer MP. Global Impact of Coronavirus Disease 2019 Infection Requiring Admission to the ICU: A Systematic Review and Meta-analysis. Chest 2021;159(2):524-36. doi:https://doi.org/10.1016/j.chest.2020.10.014

[14] Klein SJ, Mayerhöfer T, Fries D, Preuß Hernández C, Joannidis M, Collaborators, et al. Elevated HbA1c remains a predominant finding in severe COVID-19 and may be associated with increased mortality in patients requiring mechanical ventilation. Critical Care 2021;25(1):1-4. doi:10.1186/s13054-021-03730-2

[15] Memon B, Abdelalim EM. ACE2 function in the pancreatic islet: Implications for relationship between SARS-CoV-2 and diabetes. Acta Physiologica 2021;233(4):1-13. doi:10.1111/apha.13733

[16] Larsson E, Brattstrom O, Agvald-Ohman C, Grip J, Jalde FC, Stralin K, et al. Characteristics and outcomes of patients with COVID-19 admitted to ICU in a tertiary hospital in Stockholm, Sweden. Acta anaesthesiologica Scandinavica 2021;65(1):76-81. doi:10.1111/aas.13694

[17] Rysz S, Jonsson Fagerlund M, Rimes-Stigare C, Larsson E, Campoccia Jalde F, Mårtensson J. Chronic dysglycemia and risk of SARS-CoV-2 associated respiratory failure in hospitalized patients. Acta anaesthesiologica Scandinavica 2022;66(1):48-55. doi:10.1136/bjsports-2021-104080.

[18] Landstra CP, de Koning EJP. COVID-19 and Diabetes: Understanding the Interrelationship and Risks for a Severe Course. Frontiers in endocrinology 2021;12:649525. doi:10.3389/fendo.2021.649525

For peer review only - http://bmjopen.bmj.com/site/about/guidelines.xhtml

[19] Wang S, Ma P, Zhang S, Song S, Wang Z, Ma Y, et al. Fasting blood glucose at admission is an independent predictor for 28-day mortality in patients with COVID-19 without previous diagnosis of diabetes: a multi-centre retrospective study. Diabetologia 2020;63(10):2102-11. doi:10.1007/s00125-020-05209-1
[20] Holman N, Knighton P, Kar P, O'Keefe J, Curley M, Weaver A, et al. Risk factors for COVID-19-related mortality in people with type 1 and type 2 diabetes in England: a population-based cohort study. The lancet Diabetes & endocrinology 2020;8(10):823-33. doi:10.1016/S2213-8587(20)30271-0
[21] Dennis JM, Mateen BA, Sonabend R, Thomas NJ, Patel KA, Hattersley AT, et al. Type 2 Diabetes and COVID-19-Related Mortality in the Critical Care Setting: A National Cohort Study in England, March-July 2020. Diabetes Care 2021;44(1):50-7. doi:10.2337/dc20-1444
[22] Cariou B, Hadjadj S, Wargny M, Pichelin M, Al-Salameh A, Allix I, et al. Phenotypic characteristics and prognosis of inpatients with COVID-19 and diabetes: the CORONADO study. Diabetologia 2020;63(8):1500-15. doi:10.1007/s00125-020-05180-x

Figure legends

Figure 1. Flow chart of study population

Figure 2. Prevalence of prediabetes, unknown diabetes and known diabetes among 206 consecutive ICU patients with Covid-19

Figure 3. Probability of survival in no chronic dysglycemia patients and in patients with chronic dysglycemia

For peer review only

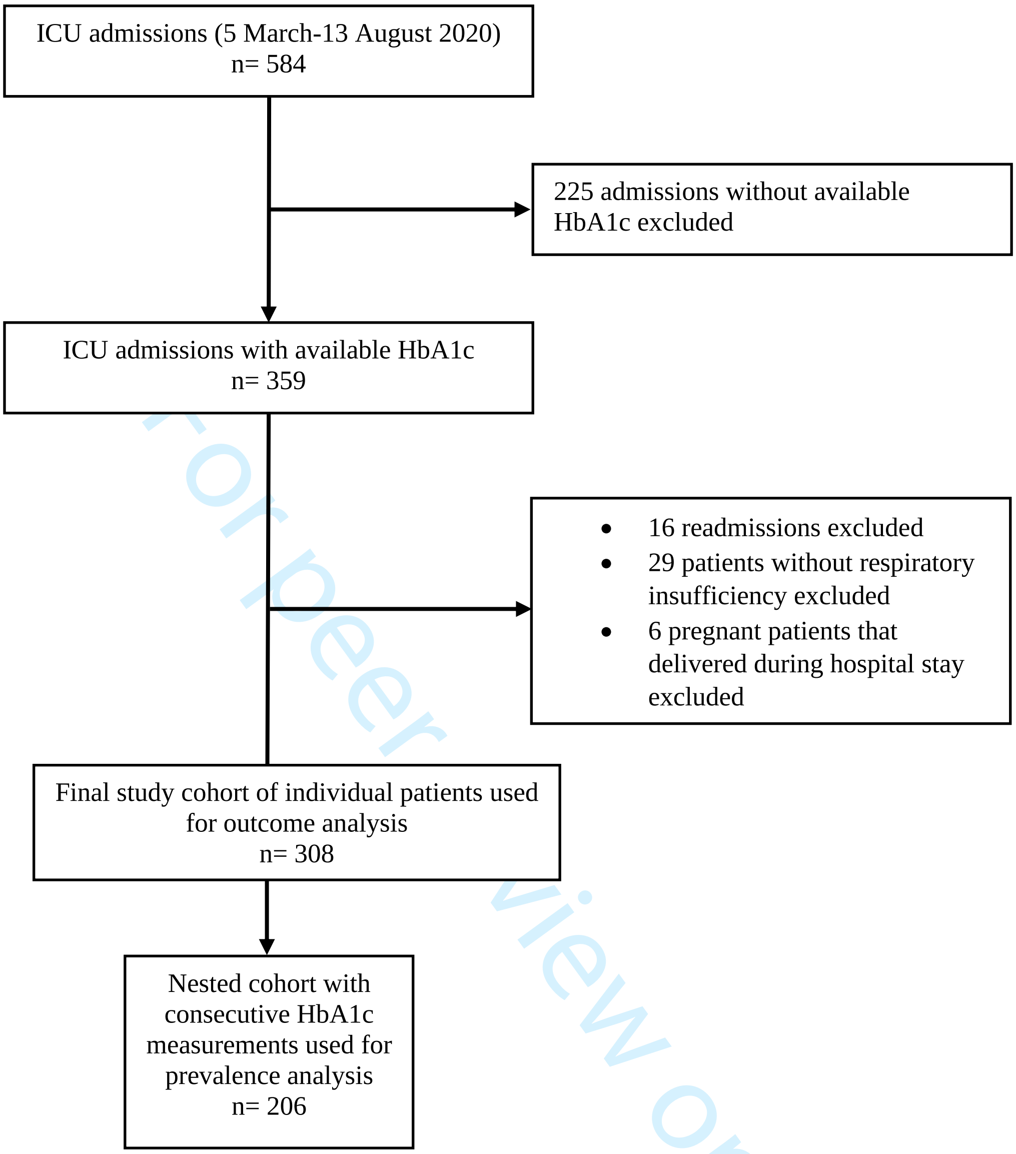

For peer review only

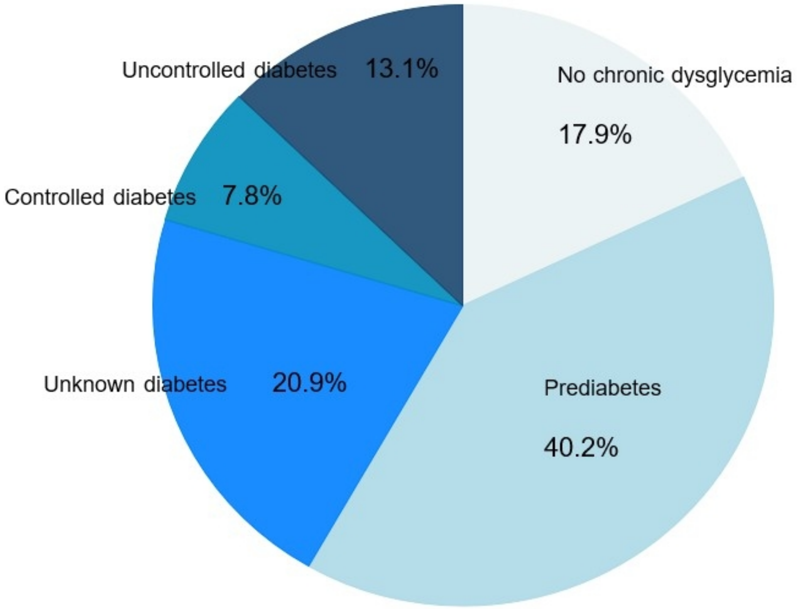

Figure 2. Prevalence of prediabetes, unknown diabetes and known diabetes among 206 consecutive ICU patients with Covid-19

144x102mm (144 x 144 DPI)

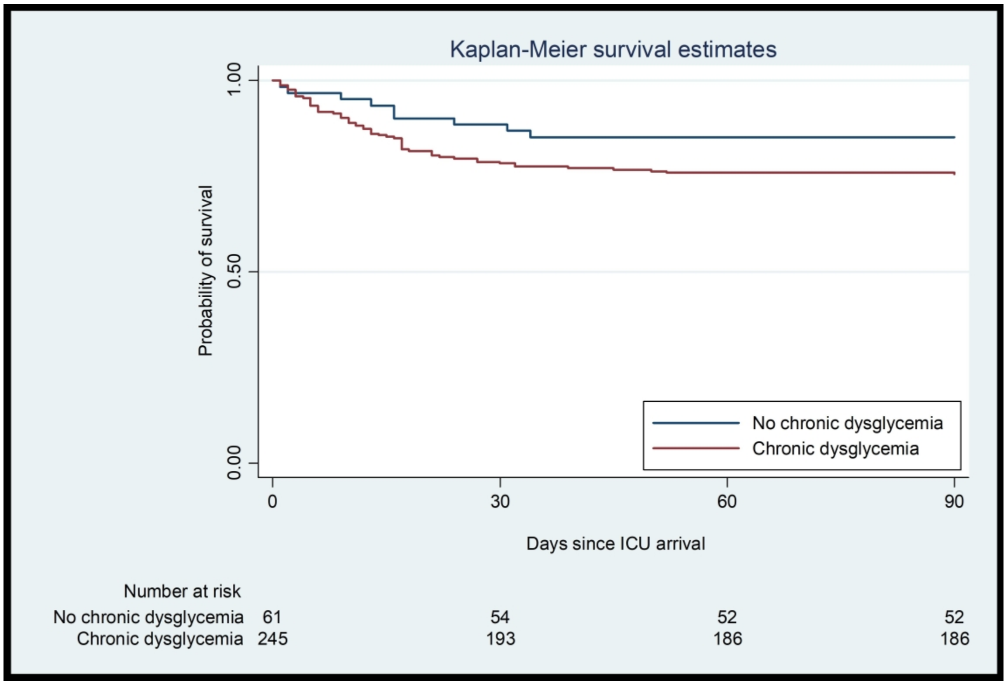

Figure 3. Probability of survival in no chronic dysglycemia patients and in patients with chronic dysglycemi

770x521mm (144 x 144 DPI)

Table S1: Post-hoc exploratory comparison between the subgroups for 90 days mortality and Renal replacement therapy

**Subgroups**	**90 day mortality**		**Renal replacement therapy**	
	N (%)	p	N (%)	p
No chronic dysglycemia vs Prediabetes	9/61 (14.7) vs 28/114 (24.5)	0.17	17/61 (27.8) vs 22/114 (19.2)	0.25
No chronic dysglycemia vs Unknown diabetes	9/61 (14.7) vs 12/60 (20.0)	0.48	17/61 (27.8) vs 11/60 (18.3)	0.28
No chronic dysglycemia vs Controlled diabetes	9/61 (14.7) vs 7/25 (28.0)	0.22	17/61 (27.8) vs 1/25 (4.0)	0.01
No chronic dysglycemia vs Uncontrolled diabetes	9/61 (14.7) vs 15/48 (31.2)	0.06	17/61(27.8) vs 8/48 (16.6)	0.25
Prediabetes vs Unknown diabetes	28/114 (24.5) vs 12/60 (20.0)	0.57	22/114 (19.2) vs 11/60 (18.3)	1
Prediabetes vs Controlled diabetes	28/114 (24.5) vs 7/25 (28.0)	0.79	22/114 (19.2) vs 1/ 25 (4.0)	0.07
Prediabetes vs Uncontrolled diabetes	28/114 (24.5) vs 15/48 (31.2)	0.43	22/114 (19.2) vs 8/48 (16.6)	0.8
Unknown diabetes vs Controlled diabetes	12/60 (20.0) vs 7/25 (28.0)	0.41	11/60 (18.3) vs 1/25 (4.0)	0.1
Unknown diabetes vs Uncontrolled diabetes	12/60 (20.0) vs 15/48 (31.2)	0.18	11/60 (18.3) vs 8/48 (16.6)	1
Controlled vs Uncontrolled diabetes	7/25 (28.0) vs 15/48 (31.2)	0.77	1/25 (4.0) vs 8/48 (16.6)	0.01
No chronic dysglycemia and prediabetes vs unknown and known diabetes	37/175 (21.1) vs 34/133 (25.5)	0.41	39/175 (22.2) vs 20/133 (15.0)	0.14

P values calculated with Fischer’s exact test

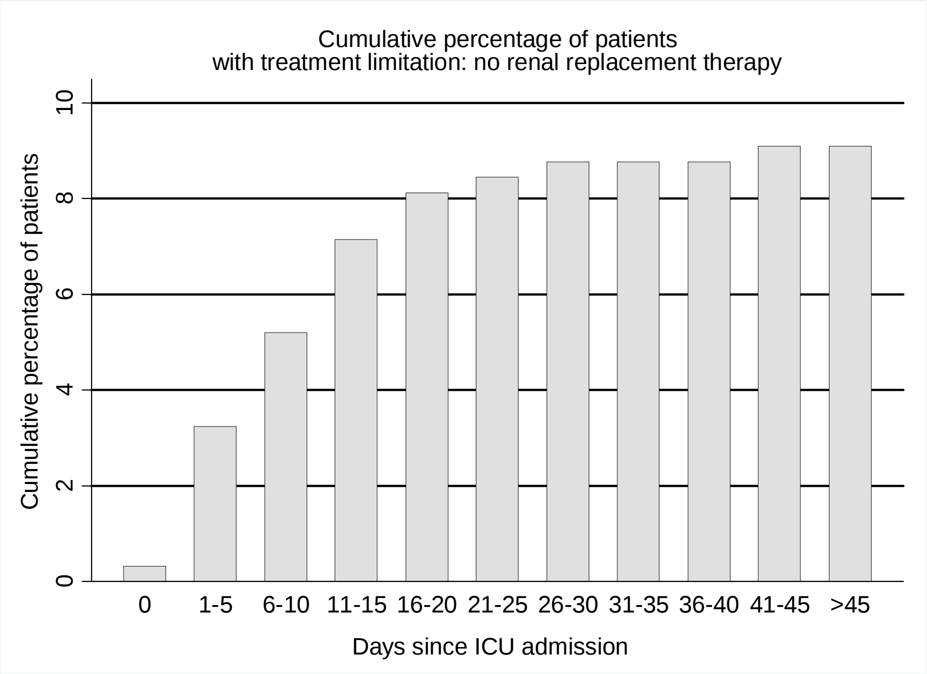

Figure S1. Cumulative percentage of patients with a treatment limitation: no renal replacement therapy. Day 0 (zero) denotes the day of admission to the intensive care unit.

For peer review only - http://bmjopen.bmj.com/site/about/guidelines.xhtml

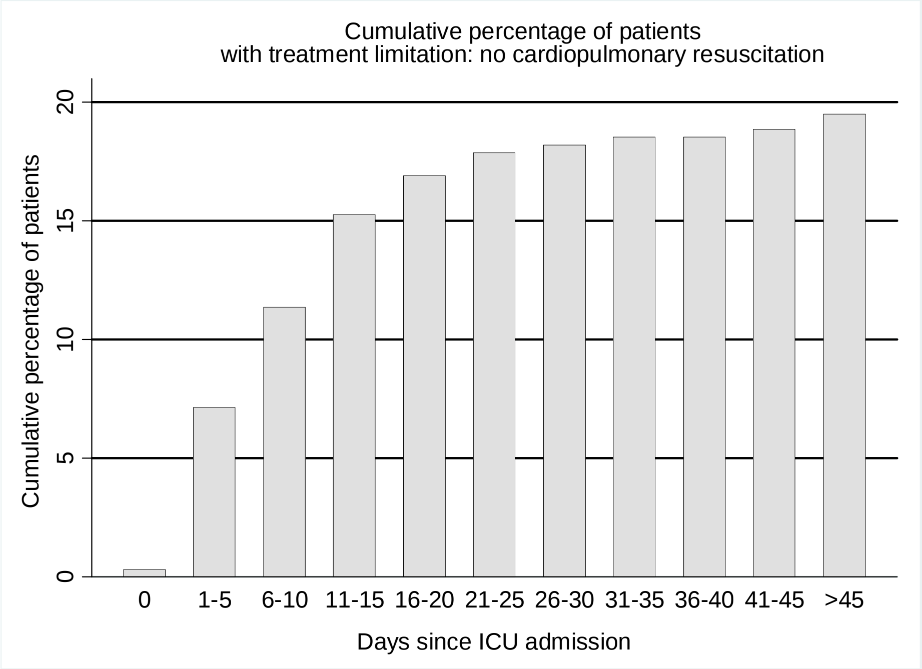

Figure S2. Cumulative percentage of patients with a treatment limitation: no cardiopulmonary resuscitation. Day 0 (zero) denotes the day of admission to the intensive care unit.

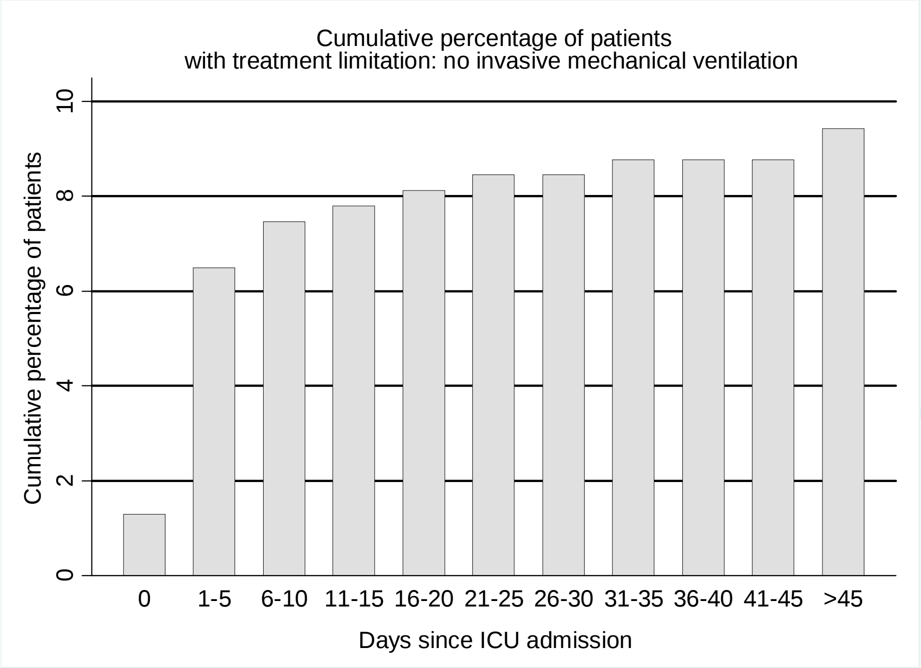

Figure S3. Cumulative percentage of patients with a treatment limitation: no invasive mechanical ventilation. Day 0 (zero) denotes the day of admission to the intensive care unit.

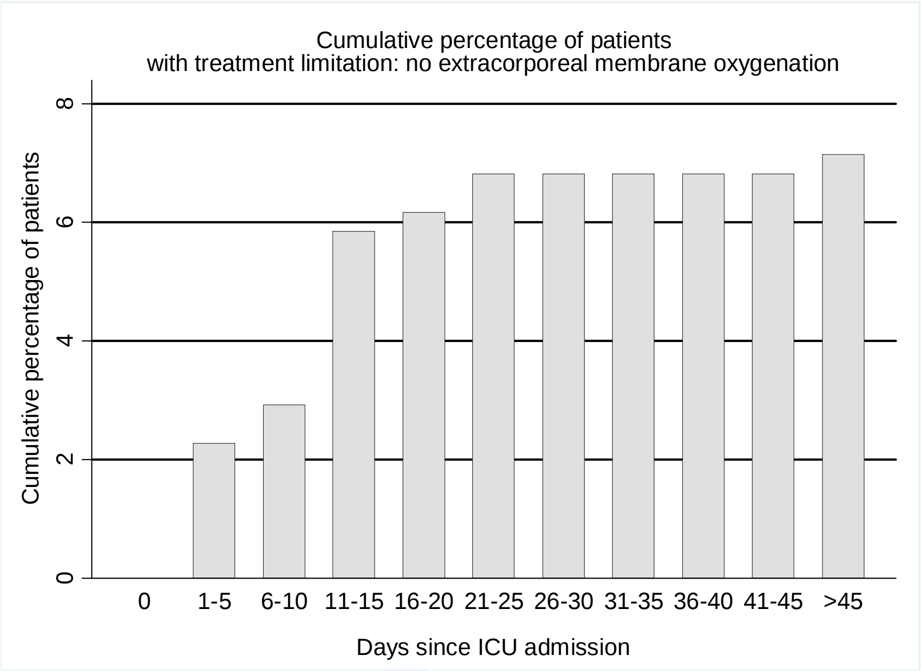

Figure S4. Cumulative percentage of patients with a treatment limitation: no extracorporeal membrane oxygenation. Day 0 (zero) denotes the day of admission to the intensive care unit.

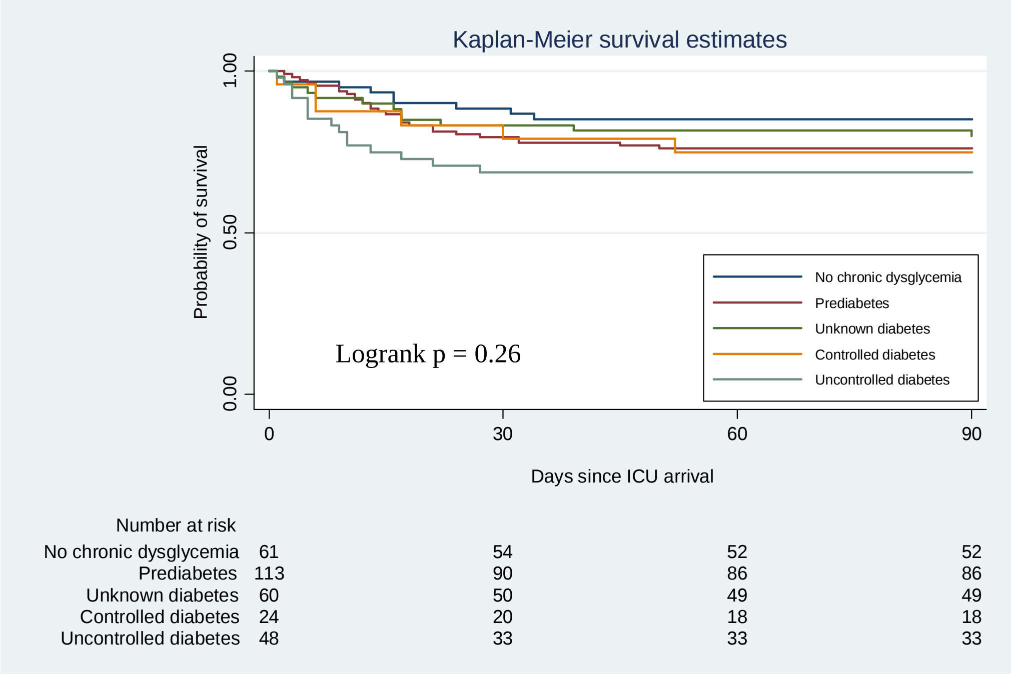

Figure S5. Probability of survival in the study groups

STROBE Statement—Checklist of items that should be included in reports of ***cohort studies***

Page references for document: **Chronic dysglycemia in critically ill patients with Covid-19 – a retrospective cohort study**

	Item No	Recommendation	Page No
**Title and abstract**	1	(*a*) Indicate the study's design with a commonly used term in the title or the abstract	1
		(*b*) Provide in the abstract an informative and balanced summary of what was done and what was found	2-3
**Introduction**			
Background/rationale	2	Explain the scientific background and rationale for the investigation being reported	3-4
Objectives	3	State specific objectives, including any prespecified hypotheses	4
**Methods4**			
Study design	4	Present key elements of study design early in the paper	4, 5
Setting	5	Describe the setting, locations, and relevant dates, including periods of recruitment, exposure, follow-up, and data collection	5-6
Participants	6	(*a*) Give the eligibility criteria, and the sources and methods of selection of participants. Describe methods of follow-up	5-7
		(*b*) For matched studies, give matching criteria and number of exposed and unexposed	NA
Variables	7	Clearly define all outcomes, exposures, predictors, potential confounders, and effect modifiers. Give diagnostic criteria, if applicable	7-8
Data sources/ measurement	8*	For each variable of interest, give sources of data and details of methods of assessment (measurement). Describe comparability of assessment methods if there is more than one group	5-7
Bias	9	Describe any efforts to address potential sources of bias	5
Study size	10	Explain how the study size was arrived at	5-6, Figure 1
Quantitative variables	11	Explain how quantitative variables were handled in the analyses. If applicable, describe which groupings were chosen and why	6-8
Statistical methods	12	(*a*) Describe all statistical methods, including those used to control for confounding	7
		(*b*) Describe any methods used to examine subgroups and interactions	7
		(*c*) Explain how missing data were addressed	8
		(*d*) If applicable, explain how loss to follow-up was addressed	NA
		(*e*) Describe any sensitivity analyses	NA
**Results**			
Participants	13*	(a) Report numbers of individuals at each stage of study—eg numbers potentially eligible, examined for eligibility, confirmed eligible, included in the study, completing follow-up, and analysed	8-9, Figure 1
		(b) Give reasons for non-participation at each stage	Figure 1
		(c) Consider use of a flow diagram	Figure 1

For peer review only - http://bmjopen.bmj.com/site/about/guidelines.xhtml

Descriptive data	14*	(a) Give characteristics of study participants (eg demographic, clinical, social) and information on exposures and potential confounders	8-9 Table 1
		(b) Indicate number of participants with missing data for each variable of interest	Table 1
		(c) Summarise follow-up time (eg, average and total amount)	NA
Outcome data	15*	Report numbers of outcome events or summary measures over time	10-11 Table 2-3

For peer review only

Main results	16	(*a*) Give unadjusted estimates and, if applicable, confounder-adjusted estimates and their precision (eg, 95% confidence interval). Make clear which confounders were adjusted for and why they were included<br>(*b*) Report category boundaries when continuous variables were categorized<br>(*c*) If relevant, consider translating estimates of relative risk into absolute risk for a meaningful time period	10-11 Table 3
Other analyses	17	Report other analyses done—eg analyses of subgroups and interactions, and sensitivity analyses	11 Figure 2,3,S1-4, S5
**Discussion**			
Key results	18	Summarise key results with reference to study objectives	11
Limitations	19	Discuss limitations of the study, taking into account sources of potential bias or imprecision. Discuss both direction and magnitude of any potential bias	14
Interpretation	20	Give a cautious overall interpretation of results considering objectives, limitations, multiplicity of analyses, results from similar studies, and other relevant evidence	15
Generalisability	21	Discuss the generalisability (external validity) of the study results	13-14
**Other information**			
Funding	22	Give the source of funding and the role of the funders for the present study and, if applicable, for the original study on which the present article is based	15

*Give information separately for exposed and unexposed groups.

Note: An Explanation and Elaboration article discusses each checklist item and gives methodological background and published examples of transparent reporting. The STROBE checklist is best used in conjunction with this article (freely available on the Web sites of PLoS Medicine at http://www.plosmedicine.org/, Annals of Internal Medicine at http://www.annals.org/, and Epidemiology at http://www.epidem.com/). Information on the STROBE Initiative is available at http://www.strobe-statement.org.

For peer review only
